# Process and Structure Modeling of Architected Thermoplastic Composites Using Shape Forming Elements

**DOI:** 10.3390/polym18091098

**Published:** 2026-04-30

**Authors:** Rebecca H. Olanrewaju, Yuefeng Jiang, Thao D. Nguyen, David O. Kazmer

**Affiliations:** 1 Francis College of Engineering, University of Massachusetts Lowell, Lowell, MA 01854, USA; david_kazmer@uml.edu; 2 Whiting School of Engineering, Johns Hopkins University, Baltimore, MD 21218, USA

**Keywords:** coextrusion, shape forming elements, architected composites, liquid crystalline polymer, polyamide, die design, regression modeling

## Abstract

Architected polymer composites use spatially organized phases to achieve targeted property combinations. Shape forming elements (SFEs) are modular coextrusion die inserts that impose internal architectures by reshaping multiple melt streams. This study evaluates three SFE designs (Jacks, I-Beam, and Barn Door) that position a liquid crystalline polymer (LCP) and an amorphous polyamide (APA) in distinct core–shell configurations. Polymer clay prototyping and ANSYS Polyflow simulations were used to screen flow behavior, followed by extrusion at two puller speeds and characterization via optical microscopy and tensile testing. Microscopy revealed that abrupt area transitions and viscosity contrast disrupt encapsulation and distort designed features. Regression analysis showed that LCP content governs stiffness and strength, while higher puller speed enhances reinforcement through molecular orientation. Cross sectional geometries were quantified using interfacial perimeter, moments of inertia, and polar dispersion ratios, and correlated to tensile performance. Increased interfacial length reduced modulus, strength, and ductility. Modulus improved with LCP orientation and confinement, strength increased when LCP was placed at vertical extremities, and elongation was maximized by horizontally distributing LCP within a thick APA shell. These results demonstrate that SFEs enable tunable tradeoffs between stiffness, strength, and ductility.

## 1. Introduction

Architected polymer composites have attracted increasing interest due to their ability to combine lightweight design with tailored mechanical performance through controlled internal structure. Unlike conventional polymer blends, where microstructure is governed largely by material compatibility and processing conditions, architected composites enable deliberate spatial organization of multiple phases to achieve targeted property combinations. Coextrusion provides a more promising route for producing such composites because it allows continuous manufacturing of multi material structures.

Among the material systems used in architected composites, liquid crystalline polymers (LCPs) represent a unique class of thermoplastics due to their ability to form highly ordered molecular domains under flow. This behavior leads to exceptional stiffness and strength among the direction of alignment, while also introducing pronounced anisotropy in mechanical properties. Unlike conventional amorphous or semicrystalline polymers, thermotropic LCPs exhibit liquid crystalline phases in the melt that enable rapid development of molecular orientation under shear and extensional flow, making their final properties highly dependent on processing history [1,2].

From a rheological standpoint, LCPs are characterized by relatively low melt viscosity, strong shear thinning behavior, and complex viscoelastic responses associated with domain alignment and relaxation. These features can complicate processing, particularly in immiscible blends with conventional thermoplastics, where differences in rheological behavior influence material morphology and stability during flow. During extrusion, LCP phases can evolve from dispersed domains into highly oriented fibrillar or continuous structures depending on processing conditions such as shear rate, temperature, and draw ratio. The formation and alignment of these structures play a critical role in determining mechanical performance, as highly oriented LCP domains can significantly enhance stiffness and strength, whereas poorly aligned morphologies are often associated with reduced performance [3,4,5].

As a result, the behavior of LCP containing systems is highly sensitive to both processing conditions and the evolution of flow induced morphology. These complexities have motivated the development of modeling approaches, including computational flow dynamics and structure property modeling to better predict material behavior during polymer processing and guide the design of extrusion systems. However, accurately capturing the coupling between flow induced orientation, evolving morphology, and resulting mechanical performance remains a significant challenge, partially in extrusion processes where spatial control of material placement is limited [6,7,8].

Traditional coextrusion, including layer multiplying elements (LMEs), primarily create uniform layered architectures by repeatedly splitting and stacking multiple material streams. While LMEs are effective for high interfacial area, these methods are limited in their ability to create non uniform or geometry dependent internal arrangements. More recent efforts have explored flow manipulation strategies aimed at producing complex cross-sectional architectures by redirecting melt streams within specially designed dies. However, these operation-based approaches often rely on indirect control of flow behavior, making the resulting structures very highly sensitive to rheological differences, processing conditions, and minor geometric variations [9]. Shape forming elements (SFEs) were previously introduced as modular coextrusion dies capable of manipulating internal material arrangements through tailored flow paths. Earlier work demonstrated the ability of SFEs to generate complex internal architectures and influence composite mechanical behavior. At the same time, that study highlighted important limitations, including shape fidelity, sensitivity to processing conditions, and discrepancies between simulation predictions, polymer clay prototypes, and experimental extrusion results. These findings suggested that relying primarily on abstract flow operations may be insufficient for achieving reliable and reproducible architectural control [10]. Building on these observations, the present work adopts a geometry enforced SFE design methodology that prioritizes direct control of material placement within the die rather than indirect flow steering. In this framework, internal channels physically constrain material pathways to guide the formation of predefined cross-sectional architectures. This approach enables systematic investigation of how die geometry, material placement, and processing parameters influence resulting microstructure and mechanical performance.

Mechanical performance in immiscible polymer composites is closely tied to material morphology and interfacial interactions. Interfacial tension and viscosity ratios govern the stability of dispersed or co-continuous morphologies and influence how phases redistribute. In LCP containing composites, reinforcement efficiency depends not only on the formation of fibrillar domains but also on their alignment and spatial distribution within the cross section. Oriented LCP structures can significantly increase modulus and stress at break, whereas poorly oriented or sheet-like morphologies are associated with brittle fracture and reduced strain to failure [11]. These observations highlight the importance of controlling both flow history and architectural placement of reinforcing phases when designing coextruded composites.

Based on this framework, the scope of the present study is to investigate the role of geometry enforced shape forming element dies in controlling material placement and resulting mechanical performance in coextruded architected composites. The study focuses on simulation and prototype driven design to evaluate how predefined internal architectures and material configurations, particularly in the placement of liquid crystalline polymer within the composite cross section, influence modulus, stress at break, and strain to failure. Emphasis is placed on understanding structure property relationships arising from controlled geometry rather than modifying interfacial chemistry, and this compatibilizers are not considered. This work aims to establish design guidelines for achieving targeted mechanical properties through geometric control of material architecture.

Shape forming elements (SFEs) provide a versatile platform for implementing such architectural control within coextrusion dies. These modular components enable manipulation of multiple melt streams through tailored internal geometries, allowing for controlled redistribution and organization of material placement within the cross section. While earlier implementations demonstrated the ability to generate complex internal structures, achieving consistent and predictable architectures remained a challenge due to sensitivity to flow conditions and material behavior. More recent developments have therefore focused on leveraging geometric constraints within the die to directly guide material placement, providing a more robust pathway for establishing reproducible structure property relationships in architected polymer composites.

## 2. Materials and Methods

### 2.1. Shape Forming Elements

SFEs are modular coextrusion die components designed to manipulate multiple melt streams during coextrusion processing. SFE designs can implement a range of flow “operations,” including material inversion (swapping melt positions), rotational reorientation (changing axial/circumferential alignment), and segmentation/cutting (splitting the flow into multiple regions). SFEs can also selectively reposition portions of the flow to redistribute material within the cross section, and replication concepts can duplicate existing arrangements to increase architectural complexity without adding extruders. Together, these operations provide a flexible framework for generating nontraditional composite architectures with controlled internal features. Prior SFE designs relied on iterative melt manipulations to create complex architected composites [10]. While this approach was conceptually feasible, the extruded cross sections were highly sensitive to processing conditions, and the prototyping and simulations conducted did not match the experimental results.

Based on these observations, the present work adopts a fundamentally different SFE design philosophy centered on direct geometric enforcement of composite architectures within the die. Rather than prioritizing the execution of the more abstract flow operations, the new methodology integrates solid channel shaping, converging flow constraints, and material separating features to physically constrain the polymer melts into predefined cross sectional configurations during processing [12,13,14]. These design elements are used to restrict lateral material migration, provide additional stability to material interfaces, and regulate local deformation pathways as the melt flows through the shaping region [15,16,17]. By constraining both the material position and flow development, the die geometry actively controls the evolution of the internal architecture rather than passively redirecting the flow trajectories of materials [18,19,20,21,22]. As a result, this methodology enables a more predictable coupling between die geometry, and resulting composite cross sections.

**Figure 1:**
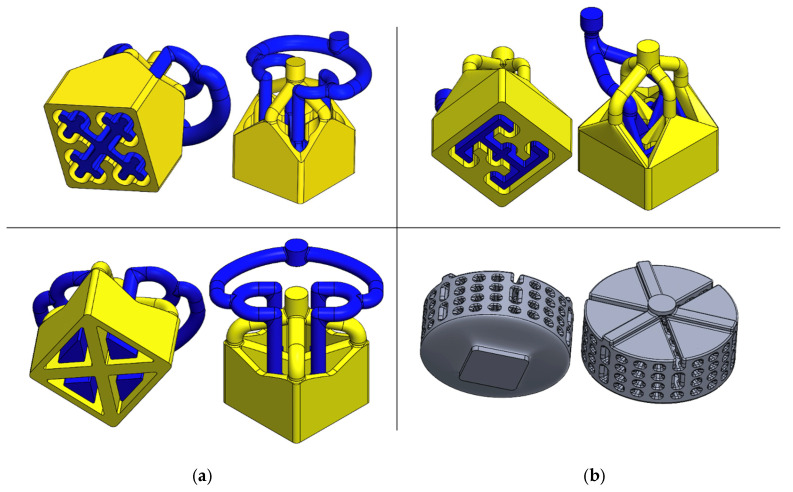
SFE flow path designs: (**a**) top, “Jacks”, bottom, “Barn Door”; (**b**) top, “I-Beam”, bottom, “Mixer Die”.

Figure 1 illustrates the flow paths in three SFE designs, as well as a Mixer Die design that are all contained within modular dies for use on a co-extruder as later described. The “Jacks” SFE (panel a, top) provides an outlet face with interlocking “Jacks”-like internal channels having four lobed arms with rounded ends; the internal manifolds split and route the two streams into multiple curved paths before they recombine at the exit, aiming to create a multi-armed internal architecture with an extensive and interlocking interface. The “I-Beam” SFE (panel b, top) provides a vertical web connecting two horizontal flanges that are formed by internal channels guiding one material stream into the I-shaped region while the other occupies the surrounding space, targeting an I-beam-style reinforcement architecture. The “Barn Door” SFE (panel a, bottom) provides a thick cross-shaped “X” divider defining four triangular “window” regions; the flow routing is meant to place material into those four regions (or around the cross), producing a framed, compartmentalized cross section. For comparison purposes, a Mixer Die (panel b, bottom) was designed with multiple levels of radial grooves connected by a matrix of vertical drops to repeatedly divide and recombine the melt stream for distributive or dispersive mixing.

By directly contrasting the Mixer Die with the neat materials and geometry enforced SFEs, this study highlights two fundamentally different strategies for manipulating polymer systems, indirect flow manipulation through repeated mixing operations and direct architectural control through physical geometric constraint. While mixer dies are effective at promoting material interaction and homogenization, they offer limited control over the final internal architecture and are highly sensitive to material properties and processing conditions. In contrast, the geometry-enforced SFE approach constrains both material position and flow evolution, reducing sensitivity to process variability and improving reproducibility across simulation, polymer clay prototyping, and experimental extrusion. The inclusion of the Mixer Die therefore provides both a processing benchmark and a conceptual contrast that clarifies the advantages of geometry-enforced shaping when the objective is deliberate internal architecture formation rather than bulk material mixing.

### 2.2. Materials

The material system utilized in this work consisted of a liquid crystalline polymer (LCP) as a reinforcement phase that is coextruded with a transparent microcrystalline or “amorphous” polyamide (APA) commonly used in optical and high-performance ballistic applications. This APA, Trogamid CX7323 (Evonik Industries AG, Essen, Germany), is composed of cycloaliphatic diamine units and long chain aliphatic diacid segments within the polymer backbone that together suppress large scale crystallite formation. As a result, the material exhibits optical transparency due to the presence of crystallites that are sufficiently small to avoid significant light scattering [23].

The LCP (liquid crystalline polymer) material was Vectra A950 (Celanese Corporation, Irving, TX, USA), which provides high stiffness, thermal stability, and ability to form highly oriented fibrillar morphologies under extensional and shear flow conditions [2,5,18,24,25,26]. Vectra A950 is an aromatic thermotropic liquid crystalline polymer that is characterized by a rigid rod like molecular structure that promotes strong molecular orientation during melt processing. Under the appropriate flow conditions, the material form fibrillar microstructure that aligns with the direction of flow, enabling the material to function as an in situ reinforcement when embedded within a compatible thermoplastic matrix, while its thermal stability supports processing alongside other engineering polymers such as polyamides [27,28,29].

In addition to material selection, the placement of each polymer within the core or shell of the coextrudate was also treated as an additional design parameter, as it directly affects both the processing induced microstructure development as well as the macroscopic mechanical performance. During coextrusion, the relative positioning of the LCP and APA phases determines the local shear and extensional flow histories that are experienced by each material, which in turn influence fibril formation and interfacial morphology. The behavior is further determined by the rheological contrast between the two materials [30,31,32,33]. Under typical processing conditions, thermotropic LCPs exhibit significantly lower apparent melt viscosity and more pronounced shear thinning behavior than the APA [34,35,36]. As a result, the LCP acts as the more mobile and less flow dominant material within the system, while the higher viscosity polyamide exerts greater resistance to deformation and dominates the flow distribution within the die [37,38]. In regions of strong velocity gradients, the lower viscosity LCP is more likely to be stretched, displaced, or encapsulated by the polyamide. Such behavior can lead to material migration and partial engulfment of the reinforcing LCP phase [39,40,41]. When the LCP material is positioned near the exterior of the cross section, the material experiences elevated shear rates due to both proximity to the die walls and the rough texture of the SFE [18]. This increases the susceptibility to interfacial distortion driven by the viscosity contrast and any secondary flow components present. These can locally redistribute the reinforcement material, leading to reduced shape fidelity and uniformity across the cross section. Conversely, when the LCP is positioned within the core region, the material experiences more symmetric deformation. Core placement also reduces the tendency for material migration, leading to more predictable material placement. These placement dependent differences are not just affected by composition, but are also controlled by the interaction between the rheological differences during processing.

### 2.3. Simulation

Finite-element simulations enable estimation of the flow behavior and optimization of the polymer process settings. As a part of the SFE development process, ANSYS Polyflow 2022 R2was used to estimate the material distributions of the coextruded material systems within the SFEs with particular emphasis on how differences in material viscosity and flow characteristics influenced material interaction and redistribution. The simulation parameters and viscosity model coefficients used in this work are summarized in Appendix A. The BCY viscosity model used in this study is expressed as

(1) $$\eta \left(\dot{\gamma }\right)=\frac{\eta_{0}}{{{\left[1+(\lambda \dot{\gamma }\right)}^{a}]}^{(1-n)/a}}$$

where $\eta \left(\dot{\gamma }\right)$ is the apparent viscosity as a function of shear rate, $\dot{\gamma }$. The parameter $\eta_{0}$ represents the zero shear viscosity, which corresponds to the Newtonian viscosity plateau at low shear rates. The time constant, λ, defines the characteristic shear rate at which the material transitions from Newtonian behavior to shear thinning behavior. The power law index, n, determines the degree of shear thinning the material has, with lower values indicating greater shear thinning behavior. The Yasuda parameter, a, controls the sharpness of the transition between the Newtonian and shear thinning region, with larger values producing a more gradual and smooth transition [42,43].

The rheological behavior of the materials was modeled using a form of the BCY model to capture the shear thinning behavior. In this implementation, the Yasuda parameter (a) is fixed at a value of 2, consistent with the formulation used in ANSYS Polyflow, resulting in a simplified Carreau type expression. The model parameters were obtained using the automatic curve fitting tool within ANSYS Polyflow is based on experimental viscosity shear rate data. The fitted parameter values used in the simulations are provided in Appendix A.

The simulations for the SFEs were run using the Generalized Newtonian non-isothermal conditions within ANSYS Polyflow, which accounts for viscosity variations arising from both shear rate and temperature. This formulation is well suited for modeling polymer flow within the SFEs where local shear rates and geometry, and thermal effects can influence the viscosity and material velocity along the flow path. A no slip boundary condition was imposed at all die walls, and fully developed inlet velocity profiles were assigned for each material stream. To minimize computation time, quarter-symmetry models for each of the three SFEs of Figure 1 were developed with symmetry conditions on the two half planes as shown in Appendix A. The polymer melts were introduced as separate phases, consistent with the experimental extrusion configuration. An atmospheric pressure condition was applied at the outlet of the die. The computational mesh was generated using tetrahedral elements. A global element size of 0.1 mm was applied throughout the domain, with local refinement (0.05 mm) at the die exit to better capture material distribution and interface development. A mesh resolution setting of 3 was used to balance computational cost and accuracy. This meshing strategy was selected to ensure adequate resolution in regions critical to cross sectional results.

### 2.4. Polymer Clay Prototyping

To assist SFE concept designs and evaluate the internal flow rearrangement behavior prior to polymer coextrusion, a prototype was created consisting of a piston driven extrusion system equipped with independent material chambers for dual feed operation and a distributor plate that directs the materials to their specific channels within the SFE. This configuration enables controlled delivery of multiple materials into the die. The prototype extruder and associated die components were fabricated using fused deposition modeling (FDM) on a Stratasys F370 3D printer. The structural components were printed using PC-ABS filament (Item No. 333-6700), with QSR soluble support material (Item No. 333-63500) employed to enable the fabrication of complex internal flow channels and removable support structures. The modular design of the system allowed for the individual SFEs to be systematically evaluated for their resulting cross sections. To visualize the material flow within the SFE geometries, polymer clay of contrasting colors was used at room temperature. Two colors of Crayola Doh polymer clay, yellow (Item No. 5700153034) and blue (Item No. 50700153042) were selected to represent the core and shell regions of the resulting extrudate. This color contrast approach enabled the direct observation of material redistribution at the outlet of the SFE assembly and provided qualitative insight into the effectiveness of the shaping operation prior to thermoplastic coextrusion experiments.

### 2.5. Polymer Extrusion

**Figure 2:**
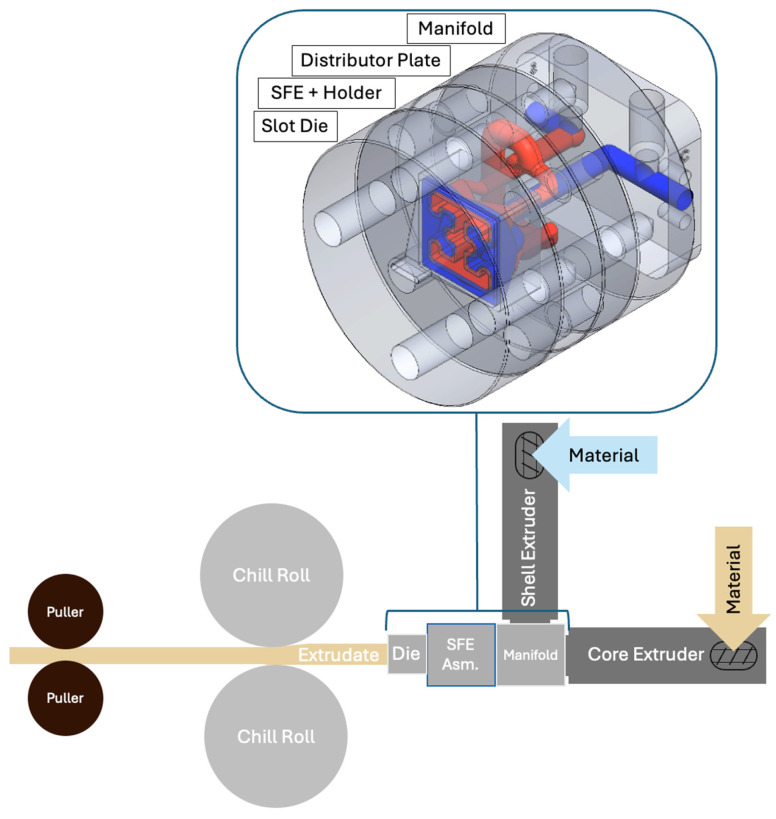
Extrusion setup.

Extrusion was carried out using a custom coextrusion system consisting of two Jugetek (Shanghai, China) single screw extruders, each having a screw length of 225 mm and a screw diameter of 20 mm. Both extruders were equipped with three independently controlled barrel temperature zones and variable screw speed control. The polymer melts generated by each extruder were conveyed through a shared manifold, shown in Figure 2, which directed the material streams into a flow distributor plate positioned upstream of the SFE. Within the manifold and distributor assembly, the two polymer melts were maintained as fully separate streams. The distributor plate routed each material into distinct inlet ports corresponding to specific flow channels within the SFE, such that the materials did not come into contact until exiting the SFE. This configuration allowed the internal geometry of the SFE to serve as the sole mechanism governing material interaction, redistribution, and final cross section result. The formed materials are then fed into a converging die plate that forces the extrudate into the form of a rounded rectangular section having a width of 10 mm and a thickness of 2 mm that is suitable for tensile testing.

The implemented processing settings are shown in Table 1. The system utilized general purpose plastication screws with a length to diameter (L/D) ratio of 10:1, capable of outputting up to 10 kg/h at 60 RPM. The barrels were smooth and non-grooved, and consisted of three independently controlled temperature zones. Both materials were processed at a nominal die temperature of 250 °C, while the barrel temperature profiles were set separately for the LCP and APA extruders. For LCP, the zone setpoints were 260/270/240 °C (Zones 1–3) to avoid over-torquing of the extruder, whereas APA used a slightly lower profile of 250/260/240 °C. The screw speed was held constant at 5 RPM during extrusion. A screw speed of 5 RPM was selected to maintain stable processing within the narrow thermal and rheological window of the materials. An OFAT (one factor at a time) study was conducted to determine suitable processing temperatures, which were set as low as possible while still enabling flow. Both materials were injection molding grade polymers, with relatively low melt strength, making them sensitive to shear and temperature fluctuations. Additionally, the extrusion system limited heat dissipation, promoting heat retention at higher screw speeds. Operation above 5 RPM also resulted in unstable motor behavior (stepper motor skipping), leading to inconsistent material feeding, the selected screw speed ensured stable processing and reproducible flow conditions. The accessible processing window was constrained by thermal and mechanical limitations, where higher temperatures let to lower melt strength and the inability to draw the coextrudate, while lower temperatures resulted in unstable extrusion due to increased melt resistance. As such, the viscosity ratio between the materials could not be independently varied over a wide range. Two puller speeds were investigated (0.51 and 1.52 cm/s) to characterize the effect of draw ratio and related effects including internal feature retention and orientation under otherwise fixed conditions. The draw ratio is defined as the area of the die outlet divided by the cross-sectional area of the drawn extrudate and varies from about 1.2 to 3.4 from the low to high puller speeds. In this study, puller speed was used as a discrete processing parameter corresponding to two controlled operating conditions rather than being directly regressed against calculated draw ratio. While draw ratio is physically related to puller speed, it is also influenced by additional factors such as melt relaxation, thermal gradients, and die swell effects. Therefore, puller speed was used as an experimentally controlled variable to ensure consistency across all specimen sets.

### 2.6. Structural Characterization

Tensile testing was conducted on specimens produced using each SFE and material configuration using an Instron 34SC-2 universal tensile testing machine. All tests were conducted at a constant crosshead speed of 5 mm/min with a gauge length of 20 mm, corresponding to an initial strain rate of approximately 0.42%/s. The tensile tests were used to characterize the elastic modulus, stress at break, and elongation to failure. The ends of each specimen were labeled and retained for imaging and further analysis.

### 2.7. Microscopy

Optical microscopy was used to evaluate the cross sections and fracture surfaces of the composite specimens. Imaging of fractured specimens was conducted to investigate deformation behavior and failure mechanisms associated with different SFEs, their draw ratios, and material configurations. Images of the cross sections were obtained using a Zeiss Discovery V20 optical stereo microscope (Oberkochen, Baden-Württemberg, Germany). The cross-section specimens were made by sectioning samples perpendicular to the primary flow direction with a Buehler Isomet 100 precision saw (Lake Bluff, IL, USA), equipped with a diamond cutting blade to minimize damage to the cross-sectional surface, material smearing, and interface distortion. All imaging was carried out under ambient laboratory conditions using a dark background to improve the contrast between the clear PA and tan LCP. The optical contrast between the two materials was sufficient for the clear identification of interface locations across the composite cross sections.

### 2.8. Image Analysis

The cross-section images of the coextruded specimens made with the SFEs were masked using a pixel editor to manually apply a white background to the cross-section images. All cropped images were then processed using a script, SFE_Image_AutoCrop_and_Scale.m, that found and measured the provided 1000-micron scale bar in pixels. This script then isolated the main cross-section in the using an intensity threshold and morphological hole-filling. By calculating region properties, the script determines the primary orientation angle of the polymer and rotates the image so its longitudinal axis is fully horizontal. Finally, the script crops the image to the object’s bounding box with a consistent 15-pixel margin, ensuring standardized, tightly cropped inputs for the subsequent image analysis.

A second script, SFE_Processed_Feature_Extraction.m, performs feature extraction by first parsing the pixel scale from the filename to establish the physical resolution in mm/pixel. The script then applies a Gaussian blur to smooth the cropped image before using the median pixel intensity of the overall polymer mask to force a 50/50 segmentation between the brighter Liquid Crystal Polymer (LCP) and darker Amorphous Polyamide (APA) phases as consistent with the blend ratio in processing. With the materials segmented, the script calculates basic macroscopic features, including cross-sectional areas, mean thickness, mean width, and the interfacial perimeter identified via morphological dilation. Afterward, the critical structural properties are computed by weighting the physical coordinates against the respective moduli of the components (6000 MPa for LCP and 1000 MPa for APA taken from subsequent tensile testing results) to estimate the location of the composite neutral axes, the principal moments of inertia, and the polar moment of inertia for each material before exporting the data and an enhanced-color visualization of the materials and interfaces. The calculations are described in a related paper and also provided in the MATLAB^TM^ script available in the Supplementary Materials [44].

### 2.9. Regression

Regression analysis was performed using the fitlm function within MATLAB^TM^ R2024b (Mathworks, Natick, MA, USA) to model the influence of processing parameters and material configuration on mechanical performance. Three modeling workflows were implemented. First, the structural behaviors (elastic modulus, stress at break, and elongation to failure) for the homogeneous materials using neat APA, a 50/50 blend of APA/LCP, and neat LCP were modeled as a function of the blend fraction and draw ratio using the script Model_Homogeneous_Behaviors.m. Second, for the SFE-made specimens, regression was performed using SFE_Fit_Model6_Legendlimits.m for factors including puller speed and material placement defined as a binary variable (with 1 indicating that the LCP material was located in the core of the cross section, and 0 indicating that APA was located in the core). Interaction terms between puller speed and LCP placement were also included to capture any coupled effects between draw induced orientation and material positioning. Separate regression analyses were conducted for each SFE geometry to isolate architecture-specific behaviors. Third, the structural data for all SFE specimens were regressed against the features characterized by the image analysis of the cross-sections. The goal of this third analysis was to evaluate the underlying structure-property relationships based purely on fundamental physics. Accordingly, given the number of potential factors, an exhaustive combinatoric approach was implemented using Model_All_Responses_Combinatorics.m to find the best subsets of determinants with three to seven factors excluding interaction effects. The results are provided in the appendix and used to guide the final selection of determinants that were evaluated using Structure_Property_Regression_Final_Factors.m. To mitigate the risk of overfitting associated with the combinatoric best subset approach model selection used the adjusted R-squared to penalize the inclusion of non-predictive variables. In addition, strict parsimony was enforced by limiting the number of retained predictors (3–6 factors), ensuring that the selected factors were statistically significant and physically meaningful. Due to the relatively modest dataset size (*N* = 63), explicit cross validation was not implemented, as partitioning the dataset would significantly reduce the effective training set size relative to the number of candidate predictors. Instead, model robustness was evaluated through consistency of physically interpretable predictors across multiple model formulations. To further assess potential overfitting, an additional set of analyses was conducted in which a universal regression model structure was enforced across all mechanical properties; these universal models exhibited reduced statistical performance, supporting the use of property specific models and indicating that the identified relationships reflect distinct underlying physical mechanisms rather than artifacts of overfitting.

In all workflows, the regression models were initially fitted to the full dataset and then evaluated using studentized residuals to identify potential outliers. Data points with residual exceeding the 95% confidence threshold were considered anomalous and removed from the dataset. The final regression models were then refitted using the cleaned data and used to generate predicted response trends along with 95% confidence intervals. The outlier removal process resulted in the exclusion of 7 data points for the elastic modulus model (*N* = 56 retained), 6 data points for the stress at break model (*N* = 57 retained), and 7 data points for the elongation to failure model (*N* = 56 retained), corresponding to approximately 10–11% of the dataset. For ease of results interpretation, the model coefficients are provided with all factors scaled to the range of [0, 1] corresponding to their minimum and maximum values across the data set.

## 3. Results and Discussion

### 3.1. Clay Prototyping

**Figure 3:**
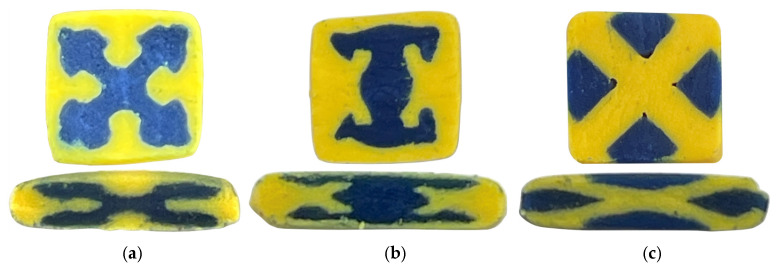
Polymer clay results for (**a**) “Jacks”; (**b**) “I-Beam”; (**c**) “Barn Door”.

The distribution of the extruded polymer clay at the outlet of the SFEs and the die are respectively provided in the top and bottom rows of Figure 3 for the different designs including “Jacks,” “I-Beam,” and “Barn Door.” The polymer clay results demonstrate that the SFE geometries successfully enforce the intended cross-sectional architectures immediately upon exiting the modular dies. For the “Jacks” design (panel a), the blue clay core clearly forms the targeted four-lobed structure with rounded extremities, fully encapsulated by the yellow matrix. The “I-Beam” SFE (panel b) effectively shapes the blue clay into a distinct web and flange configuration, demonstrating excellent control over regional material placement. In the “Barn Door” configuration (panel c), the geometry successfully compartmentalizes the blue clay into four discrete triangular quadrants separated by a distinct yellow cross-shaped divider. This confirms that the internal SFE channels accurately dictate the initial spatial arrangement of the contrasting materials prior to further downstream deformation.

As the coextruded materials pass through the downstream shaping die, the internal architectures undergo significant transverse flow and planar extension while maintaining their basic topological continuity as shown in the bottom row of Figure 3. The “Jacks” profile (panel a) experiences severe vertical compression, flattening the top and bottom lobes into the central core while stretching the lateral lobes outward into thin, elongated structures. The “I-Beam” (panel b) undergoes similar distortion with its vertical web heavily compressed into a wider central band while the upper and lower flanges are drawn out horizontally and losing their distinct distal interlocks. For the “Barn Door” (panel c), the initial “X” framework is stretched laterally, squashing the top and bottom blue compartments into thin horizontal layers and elongating the side compartments. Overall, these results illustrate how the converging material flows through the final die drastically alter the aspect ratios of the created architectures while blurring sharp corners but otherwise retaining their essential features.

The polymer clay system is used strictly as a qualitative, geometry driven visualizing material redistribution within the SFE designs. The clay does not replicate the rheological behavior, temperature dependence, interfacial tension, or viscosity ratios present in the thermoplastic coextrusion system. As a result, deviations between clay and polymer extrusion results are expected and arise from the absence of these governing physical phenomena. The primary purpose of the clay prototyping is therefore to evaluate whether the imposed geometric constraints within the SFE successfully generate the intended spatial routing of materials, rather than to provide a quantitative prediction of final polymer morphology.

### 3.2. Simulation

**Figure 4:**
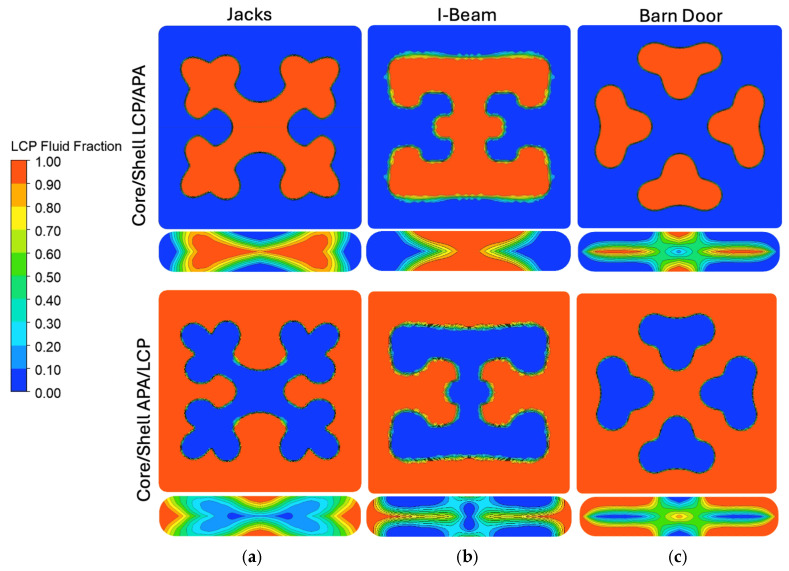
Simulation results for SFE exits and slot exits for (**a**) “Jacks; (**b**) “I-Beam”; (**c**) “Barn Door”.

ANSYS Polyflow simulations were conducted to model material flow and interface evolution within the SFEs under idealized processing conditions. The simulations provided insight into the estimated flow paths, regions of elevated shear, and the stability of material interfaces for each architecture, shown in Figure 4. For the Jacks SFE, Polyflow simulations modeled stable and symmetric redistribution of material into the arm structures. Material interfaces remained well defined, and the overall geometry of the arms was preserved throughout the simulated domain. Localized regions of increased shear were observed at the junctions where the flow divided into multiple arms, but these did not significantly disrupt the intended cross-sectional architecture. The I-Beam SFE demonstrated particularly uniform flow behavior in simulation. Polyflow results showed smooth material transport along the central web and into the flanges, with minimal interface distortion or shear localization. The absence of abrupt geometric transitions contributed to stable flow and predictable material placement, consistent with the high degree of uniformity observed in the polymer clay experiments. For the Barn Door SFE, simulations showed effective material separation but identified regions of elevated shear near sharp corners and angular transitions. These regions correspond to areas of increased deformation, indicating a higher susceptibility to interface distortion compared to the other designs. Despite this sensitivity, the simulations maintained the overall structural intent of the architecture, demonstrating that the SFE was capable of guiding material flow even under geometrically demanding conditions.

**Figure 5:**
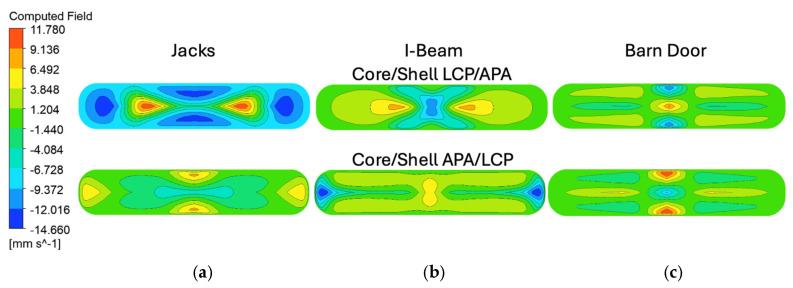
Computed field results for all slot die exits and material configurations (**a**) “Jacks; (**b**) “I-Beam”; (**c**) “Barn Door”.

Post processing of the simulation results was performed in ANSYS Polyflow, using the CFD-Post environment to visualize the interaction between the two materials as they progressed through the coextrusion die geometries, as shown in Figure 5. The fluid fraction variable, which normally ranges from 0 to 1 and represents the local volume fraction of each material, was rescaled to a range of −1 to 1 to more clearly distinguish the two materials within the flow domain. In this representation, negative values correspond to APA and the positive values correspond to LCP. A computed field was then generated by multiplying the rescaled fluid fraction by the velocity component in the primary flow direction. This computed field simultaneously encodes both material identity and flow velocity, allowing for the spatial distribution of each material and its corresponding velocity magnitude to be visualized in a single contour plot. The sign of the computed field identifies which material occupies a given region, while the magnitude reflects the local velocity of that material in the flow direction. To ensure consistent comparison between the different die geometries and material configurations, consistent color scale limits were applied across all contour plots so that the variations in the contours reflect differences in flow behavior rather than automatic rescaling of the visualization.

Figure 5 is arranged such that the columns correspond to the three die geometries while the rows correspond to the two material configurations (Core/Shell LCP/APA and Core/Shell APA/LCP). In both material configurations, the square channel sections upstream of the shaping region exhibit nearly uniform contours across all die geometries. These regions are dominated by values near zero, indicating that both materials follow similar velocity profiles as they move through the straight channel. This behavior is consistent with a fully developed channel flow in which the velocity distribution is governed primarily by the channel geometry and the no slip boundary condition at the walls, resulting in lower velocities near the walls and higher velocities toward the center of the flow domain. The similarity of these contours across all dies suggests that the upstream flow field entering the shaping region is largely independent of the downstream die geometry.

In contrast, clear differences between the dies emerge within the slot-shaped exit regions where the shaping elements redistribute the materials. For the Core/Shell LCP/APA configuration, the “Jacks” die produces highly localized regions of positive and negative values, indicating strong velocity gradients at the material interfaces as the materials are forced through intersecting flow pathways. The “I-Beam” die produces a more distributed pattern of velocity gradients, with broader regions of moderate positive and negative values across the cross-section. “The Barn Door” die exhibits a comparatively symmetric pattern with smaller velocity differences, suggesting a more uniform redistribution of the materials across the slot.

A similar trend is observed for the Core/Shell APA/LCP configuration. While the sign of the computed field reverses due to the interchange of the core and shell materials, the spatial distribution of the contours remains largely consistent for each die geometry. The “Jacks,” “I-Beam,” and “Barn Door,” produces the most localized and intense velocity gradients, but the “I-Beam” and “Barn Door” geometries distribute the velocity differences over a larger region of the cross section. These results indicate that the geometry of the shaping element primarily governs the magnitude and spatial distribution of velocity mismatches between the materials. The slot region of the die plays a dominant role in redistributing the materials and generating geometry dependent velocity gradients that may influence the deformation and stability of the architected domains during extrusion.

### 3.3. Structural Results

**Figure 6:**
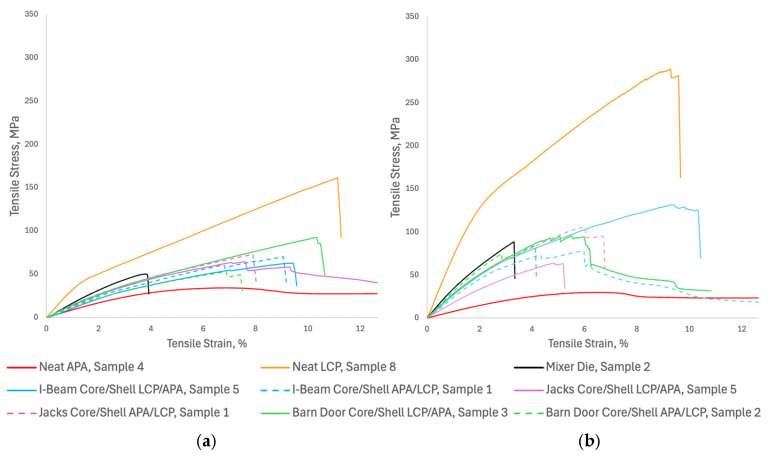
Representative tensile date for all materials and SFE configurations, (**a**) 0.51 cm/s puller speed; (**b**) 1.52 cm/s pull speed.

Figure 6 provides the representative stress-strain behavior of the baseline materials and the structured composites under puller speeds; the “representative” specimen was selected as the median stress-strain response chosen from a set of at least 5 replicate specimens produced and tested at the same conditions. The neat liquid crystal polymer (LCP, solid orange line) is the stiffest and strongest material tested albeit with a brittle failure behavior. Notably, its peak tensile stress nearly doubles from approximately 160 MPa at the 0.51 cm/s pull speed (draw ratio ~1.2) to nearly 300 MPa at 1.52 cm/s (draw ratio ~3.4), demonstrating a strong sensitivity to draw-induced molecular orientation during cooling and crystallization. In stark contrast, the neat amorphous polyamide (APA, solid red line) exhibits highly ductile yielding behavior, maintaining a low stress plateau of around 30 MPa that extends well beyond 20% strain, appearing largely unaffected by the change in draw speed. Meanwhile, the Mixer Die specimens (solid black line) fracture prematurely at roughly 3% to 4% strain in both cases, suggesting that chaotic mixing without continuous, structured reinforcement creates significant stress concentrations that lead to early, brittle failure.

The SFE-generated coextruded architectures typically lie within the mechanical performance bounds between the neat LCP and neat APA. Across the board, the higher draw ratio (1.52 cm/s) enhances the tensile strength and modulus of the composite structures, driven by the increased alignment of the reinforcing LCP phase. Furthermore, material placement plays a critical role in the structural integrity of the extrudate: configurations where LCP is placed in the core (solid blue, pink, and green lines) consistently outperform those with APA in the core (dashed lines) in both stress at break and elongation to failure. For example, at the higher pull speed, the “I-Beam” with an LCP core reaches roughly 130 MPa and exhibits a distinctive stepwise failure, whereas its APA-core counterpart fractures much earlier at approximately 80 MPa. Further insights into these behaviors are subsequently described in the next two sections investigating the image and regression analyses.

It is important to note that all tensile measurements were conducted in the extrusion direction, which represent the dominant orientation direction resulting from flow and draw induced alignment during processing. The high aspect ratio of the samples promotes strong axial alignment of the LCP material, contributing to the enhanced stiffness and strength observed at higher draw ratios. Transverse mechanical properties were not evaluated in this work; however, they are expected to differ significantly due to reduced molecular alignment and load bearing efficiency perpendicular to the flow direction. Additionally, the relatively weak interfacial adhesion between the materials is expected to further reduce transverse performance, as loading perpendicular to the flow direction promotes interfacial debonding rather than effective load transfer. These considerations highlight the inherently anisotropic nature of the coextruded composites and should be explored in future work.

### 3.4. Microscopy and Image Analysis

**Figure 7:**
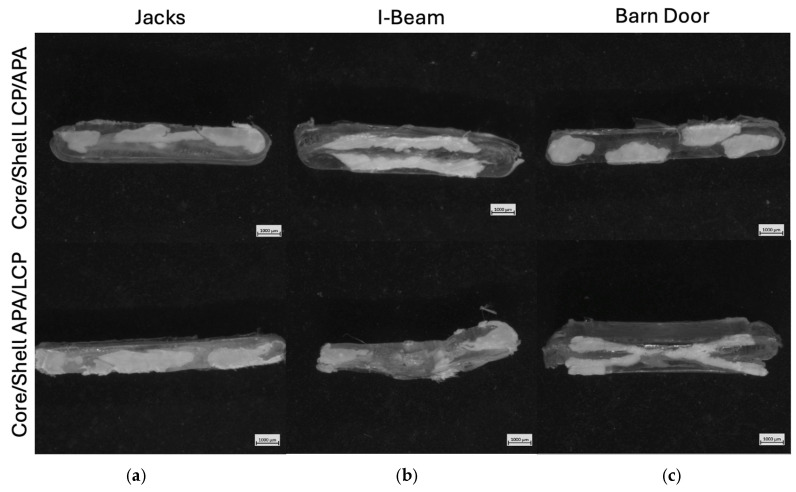
Cross sectional microscopy results for (**a**) “Jacks”; (**b**) “I-Beam”; and (**c**) “Barn Door” SFEs for a 0.51 cm/s pull speed.

The microscopy images for the “Jacks” (panel a,), “I-Beam” (panel b), and “Barn Door” (panel c) are shown in Figure 7, with the LCP-core configuration in the top row and the APA-core configuration in the bottom row. These images are from extrudates produced at the lower puller speed that resulted in larger sections that were easier to image; the images from extrudates produced at both speeds are provided in Appendix B along with image analysis results including estimated structural features. The cross sections of Figure 7 reveal strong dependencies on both die design and material placement, with less success in reproducing the intended geometries than would have been expected from either the clay prototyping or polymer processing simulation results.

For the Jacks SFE, the intended four-lobed cross-sectional geometry was largely lost in both material configurations. In the LCP/APA Core/Shell configuration (panel a, top), the low-viscosity LCP appears as a flattened, irregular central masses. While there is evidence of lateral distribution, the distinct “arms” of the “Jack” design have merged or collapsed, resulting in an elongated morphology rather than four discrete lobes. Similarly, the APA/LCP Core/Shell configuration (panel a, bottom) failed to produce the expected internal architecture. Instead, the section with the APA core is quite similar to that made with the LCP core, suggesting that any shaping from the SFE was dominated by lateral flow and interfacial tension downstream of the SFE [40,42].

The “I-Beam” SFE exhibited better geometric fidelity relative to the Jacks design, though with distinct differences between material configurations. In the LCP/APA Core/Shell configuration (panel b, top), the LCP core separated into two distinct upper and lower flanges, but the central vertical “web” connecting them is extremely thin or non-existent, indicating a break in the structure. In contrast, the APA/LCP Core/Shell configuration (panel b, bottom) produced a highly defined ‘I’ shaped region with the higher-viscosity APA core defining a vertical web and flange structure while pushing the LCP to the lateral sides of the flanges. This lack of encapsulation is consistent with the lower viscosity of the LCP, which promotes lateral spreading rather than stable confinement around the higher viscosity APA phase [18,39,41].

The “Barn Door” SFE provided the best replication of the SFE material placement. In the LCP/APA Core/Shell configuration (panel c, top), the LCP core was successfully separated into discrete regions, appearing as two dominant lateral masses rather than four distinct triangular quadrants, indicating that the horizontal “windows” from the SFE design collapsed while the vertical separation was maintained. For the APA/LCP Core/Shell configuration (panel c, bottom), the intended “X” geometry of the APA core is visible but distorted. The surrounding LCP failed to encapsulate the structure entirely, instead forming thick layers on the top and bottom surfaces while leaving the sides of the APA core relatively unconfined. This results in a layered “sandwich” morphology rather than the intended compartmentalized structure, behavior consistent with the LCP’s tendency to migrate to high-shear regions or spread laterally rather than maintain a uniform shell [7,45,46,47].

The loss of target geometry observed across several SFE designs highlights that structural fidelity is governed by a combination of geometric and material factors, rather than a single design parameter. Features such as flow symmetry, geometric sharpness, and interfacial extent all influence the stability of the evolving architecture. Asymmetric flow paths, thinner regions, and sharp corners that can introduce localized velocity gradients that promote interfacial distortion and material redistribution, while increased interfacial perimeter exacerbated instability due to the immiscibility and viscosity contrast between the phases. The “Barn Door” SFE’s geometric fidelity is attributed to its more symmetric flow configuration and reduced geometric complexity, which together promote more stable material separation. This behavior is also coupled to the rheological contrast between the LCP and APA, indicating that successful SFE design requires coordinated consideration of both geometry and material selection.

While a formal statistical analysis of shape fidelity or variance was not conducted in this study, these results highlight the role of geometric constraint in influencing material placement and structural development. Across the SFE designs, the degree of correspondence between the intended die geometry and the resulting cross section varies systematically with the imposed geometric features and the material configuration, rather than occurring randomly. This behavior is consistent with prior work on geometry enforced SFE systems, which demonstrated improved shape fidelity and reduced sensitivity to processing conditions relative to flow-manipulation-based approaches. Collectively, these observations indicate that geometric constrain provides a more reliable framework for guiding internal architecture, even in cases where full replication of the intended geometry is not achieved.

**Figure 8:**
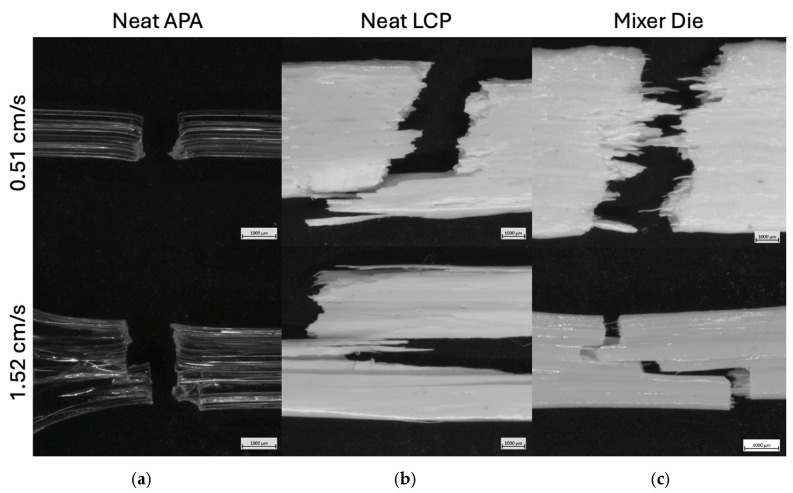
Fracture behavior of (**a**) neat APA; (**b**) neat LCP; and (**c**) Mixer Die samples.

The fracture surfaces for the neat APA, neat LCP, and 50/50 APA/LCP blend extrudates produced are respectively shown in the upper and lower rows for the Figure 8 for the respective lower and higher puller speeds. For the neat APA (panel a), both draw ratios produced fracture surfaces characteristic of a highly ductile, amorphous polymer. In both cases, the material underwent pronounced necking and substantial elongation prior to failure. The fracture surfaces are rough and irregular, consistent with extensive plastic deformation and energy dissipation before fracture. No qualitative transition in fracture mode was observed between the two puller speeds, indicating that APA maintains its tough, ductile failure behavior over the range of processing conditions investigated [23,47]. In contrast, the neat LCP (panel b) exhibited a strong dependence of fracture morphology on puller speed. At the lower draw ratio, the fracture surface is relatively smooth and planar, with little evidence of necking or plastic deformation. The fractured region appears layered, with sheet-like features rather than elongated fibrils, indicating a more brittle fracture mode. At the higher draw ratio, the fracture surface becomes significantly more complex, with the presence of elongated fibrillar features and an increased fracture surface area. This transition is consistent with increased molecular orientation induced by the higher draw rate, leading to greater load-bearing capability prior to failure and promoting fibrillation rather than catastrophic brittle fracture [18].

The Mixer Die samples (panel c) exhibit fracture behavior that is distinct from either neat material and strongly influenced by puller speed. At the lower pull speed (0.51 cm/s), the fracture surface shows a broad distribution of LCP domain sizes, ranging from relatively large regions to much finer features. The fracture surface morphology indicates partial fibrillation of the LCP phase, but without a uniform or highly aligned structure. At the higher draw ratio, the Mixer Die samples display markedly thinner and more numerous fibrils, along with a stepped fracture profile characterized by multiple fracture planes. This stepped morphology is indicative of a greater fracture surface area, indicating enhanced energy dissipation during failure. The finer fibrillar structure is consistent with increased draw-induced orientation of the LCP domains at higher pull speeds, while the stepped fracture suggests crack deflection and progressive failure rather than a single dominant fracture plane [11,18,35].

Despite the increased fibrillation and evidence of enhanced energy dissipation at higher draw ratios, the overall mechanical performance of the Mixer Die sample remains limited. The premature failure is attributed to the combined effects of morphological disorder and interfacial debonding. The highly irregular and discontinuous phase distribution introduces stress concentration sites and disrupts continuous load bearing pathways, while the increased interfacial area between the incompatible phases facilitates crack initiation and propagation along the weak interfaces.

**Figure 9:**
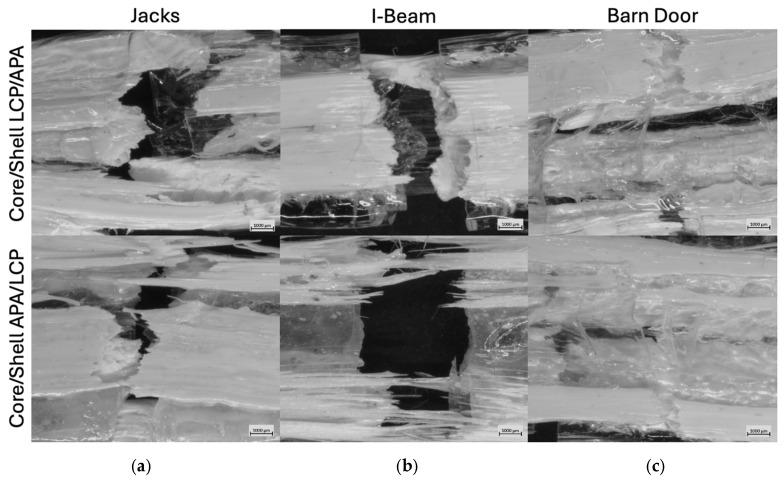
Fracture behavior of (**a**) “Jacks”; (**b**) “I-Beam”; and (**c**) “Barn Door” of all their material configurations at 0.51 cm/s pull speed.

Fracture surface microscopy of samples processed through the SFEs at the lower draw ratio are provided in the top and bottom rows of Figure 9 for LCP in the core and APA in the core, respectively. For the “Jacks” SFE, both the core/shell LCP/APA and core/shell APA/LCP configurations exhibit similar fracture surfaces dominated by regions of LCP with a wide range of domain sizes. The fracture morphology is primarily sheet-like, with only sparse and isolated fibrillar features observed. In both material configurations, fracture occurs in a brittle manner, with minimal evidence of plastic deformation. Additionally, failure is characterized by unsheathing of the LCP material from the APA region, indicating separation at the material interface during fracture.

The “I-Beam” SFE shows distinct differences between material configurations. For the core/shell LCP/APA configuration, fracture is predominantly brittle, with the fracture surface composed almost entirely of sheet-like LCP features and no observable fibrillation. Similar to the “Jacks” SFE, unsheathing of the LCP from the APA matrix is observed, suggesting limited interfacial load transfer under these processing conditions. In contrast, the “I-Beam” core/shell APA/LCP configuration exhibits a markedly different fracture morphology. In this case, a substantially higher density of fibrillar features is present within the fracture surface. These fibrils are localized to regions corresponding to areas of high shear in the SFE design, indicating enhanced deformation and orientation of the LCP material prior to fracture. While the overall failure mode remains brittle, the presence of fibrillation suggests increased resistance to crack propagation in these high-shear regions relative to other SFE geometries at the same pull speed. For the “Barn Door” SFE, both material configurations display similar fracture behavior. In each case, fracture is brittle and dominated by sheet-like LCP features rather than fibrillar pullout. Unsheathing of the LCP material is again observed as a primary failure mechanism, indicating interfacial separation between the LCP and APA phases. Despite the complex geometry of the Barn Door SFE, no significant fibrillation is observed at this lower pull speed.

**Figure 10:**
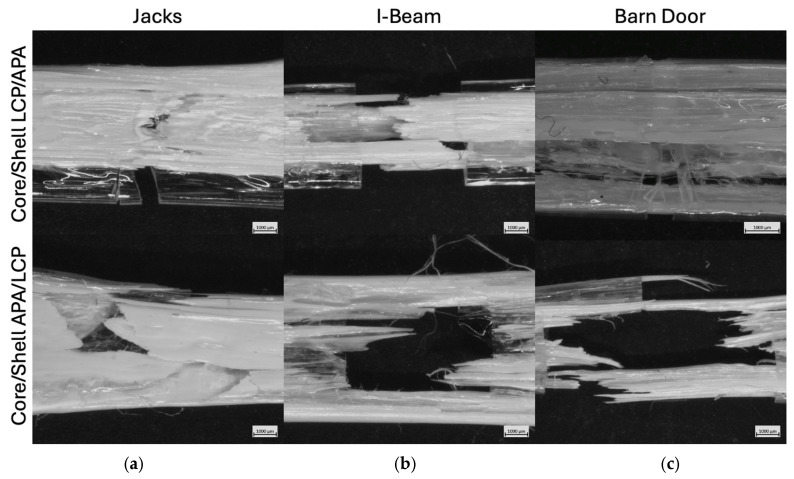
Fracture behavior of (**a**) “Jacks”; (**b**) “I-Beam”; and (**c**) “Barn Door” of all their material configurations at 1.52 cm/s pull speed.

At the higher draw ratios corresponding to a pull speed of 1.52 cm/s, fracture surface microscopy of the SFE samples in Figure 10 continues to show predominantly brittle failure across all geometries. However, increased pull speed leads to notable differences in fracture morphology, particularly for the “I-Beam” geometry and specific material configurations. For the “Jacks” SFE, both the core/shell LCP/APA and core/shell APA/LCP configurations exhibit brittle fracture with limited deformation prior to failure. In both cases, fracture surfaces show minimal complexity, with no observable fibrillar features present in the LCP phase. Some degree of unsheathing between the LCP and APA regions is observed, indicating interfacial separation during fracture, but overall fracture morphology remains relatively simple and planar compared to other SFE geometries. The “I-Beam” SFE displays the most pronounced sensitivity to both pull speed and material placement. For the core/shell LCP/APA configuration, fracture remains brittle; however, a significant degree of unsheathing is observed. The APA region exhibits a relatively clean fracture surface, while the LCP material shows a stepped fracture morphology with a larger effective fracture surface area. In addition, limited fibrillar features are present within the LCP, indicating localized deformation and orientation prior to failure. In contrast, the “I-Beam” core/shell APA/LCP configuration exhibits extensive fibrillation of the LCP material at the higher pull speed. The APA region fractures cleanly, while the LCP displays a high density of elongated fibrils, accompanied by pronounced unsheathing at the interface. This configuration shows the most complex fracture surface among all SFE samples at this pull speed, suggesting enhanced load transfer and deformation of the LCP material relative to other geometries. For the “Barn Door” SFE, fracture behavior is comparatively insensitive to pull speed. In the core/shell LCP/APA configuration, fracture remains brittle, with sheet-like LCP features dominating the fracture surface and no observable fibrillation. Some unsheathing is present, and in certain cases fracture occurs preferentially in only one of the two material phases. Similarly, the core/shell APA/LCP configuration exhibits brittle fracture with substantial unsheathing; however, in this case the LCP material predominantly fails through fibrillation rather than sheet like fracture. No direct quantitative measurement of molecular orientation was performed in this study, and no critical orientation threshold was defined. However, the observed differences in fracture morphology, particularly in the presence or absence of fibrillar features in the LCP phase, are interpreted qualitatively in the context of established processing structure relationships for LCPs. Variations in pull speed and material placement have influenced flow induced alignment during deformation, which in turn affects fibrillation behavior and crack propagation pathways.

In addition to the morphological differences observed in fracture microscopy, mechanical incompatibility between the LCP and APA phases may contribute to the interfacial failure observed in many samples. Under tensile loading, phases with different lateral contraction behaviors (as quantified by Poisson’s ratio) deform differently when constrained by an interface. This mismatch in lateral deformation can generate additional internal stresses at the interface, particularly in immiscible systems where interfacial chain entanglements are limited and interfacial strength is weak. Simulation and experimental studies of composite and blended polymer systems demonstrate that mechanical mismatches, including differences in Poisson’s ratio, can alter local stress distributions and reinforce or weaken composite behavior depending on material organization. In immiscible polymer interfaces, limited interdiffusion and poor entanglement density further reduce stress transfer capacity, making interfacial separation more likely under tensile stress. In the present coextruded composites, the increasing orientation of the LCP material at higher pull speeds may effectively change its transverse deformation behavior relative to APA, exacerbating mismatch effects and contributing to the interfacial unsheathing and progressive fracture morphologies observed [44,48,49,50,51,52].

### 3.5. Structural Behavior Modeling

#### 3.5.1. APA, LCP, and 50/50 APA/LCP Blend

**Figure 11:**
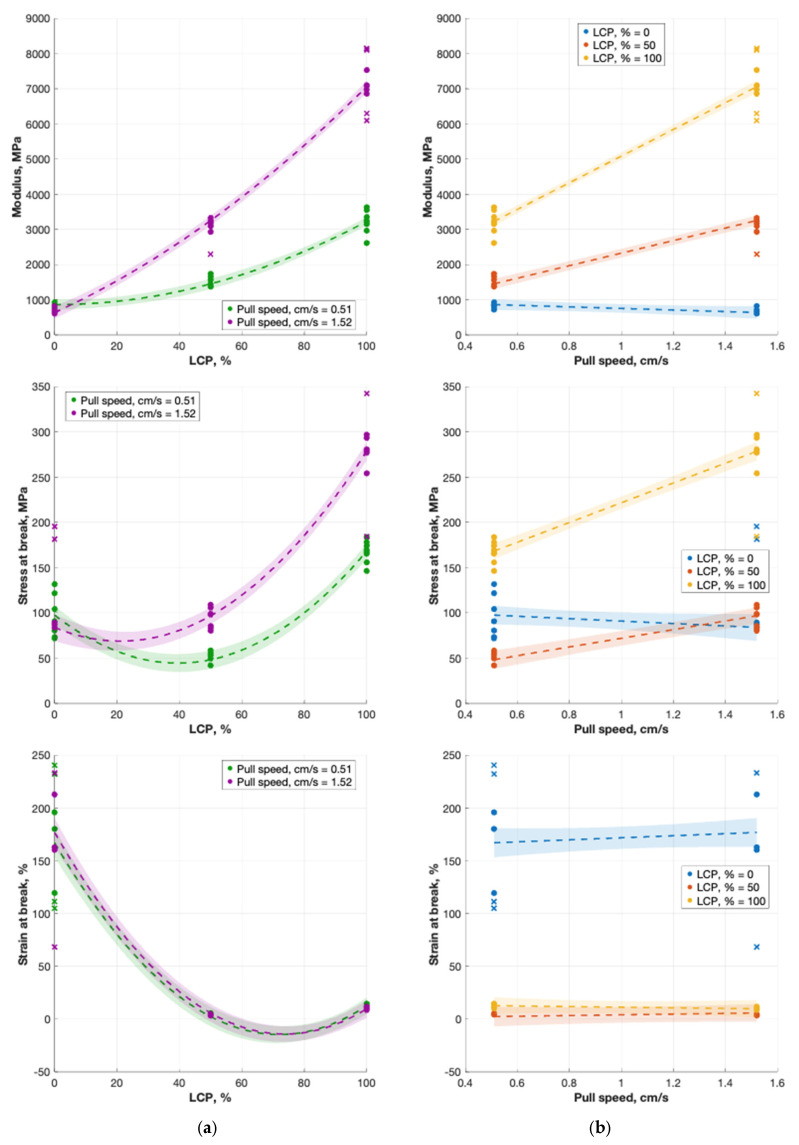
Regression analysis results for the effect of (**a**) LCP content and (**b**) pull speed on tensile properties for neat APA, neat LCP, and Mixer Die Samples.

Regression analysis was performed to evaluate how LCP content and pull speed jointly influenced the modulus, stress at break, and strain at break for the neat materials and Mixer Die samples. The resulting model coefficients are provided in Table 2, Table 3 and Table 4 with their respective responses plotted in Figure 11; the model coefficients are provided for all factors scaled in the range of [0, 1] for ease of results interpretation. All sets of results indicate strong sample composition dependent behaviors and clear interactions between composition and draw ratio, showing the role of draw induced orientation in systems containing significant LCP content [52].

The regression model for elastic modulus demonstrates a very strong fit (R2~0.991). The baseline modulus for the lesser drawn, neat APA specimens is ~976 MPa, with the modulus significantly increasing given the synergistic relationship between the LCP content and the pull speed. While increasing the pull speed slightly reduces the modulus of the baseline APA (estimate −223.7, *p* = 0.0472), the massive and highly significant interaction term (estimate 4015, *p* = 7.04 × 10^−23^) reveals that combining high draw speeds with high LCP concentrations dramatically increases the overall stiffness of the extrudate. The modulus is further increased by quadratic term for LCP (estimate 2374, *p* = 3.24 × 10^−9^) that further outweighs its linear term (estimate −2055, *p* = 1.27 × 10^−6^). At 100% LCP, the modulus for the specimens produced at the higher pull speed condition reaches approximately 7000 MPa, more than double the modulus observed for specimens produced at the lower pull speed. Specimens produced with the 50/50 blend of APA/LCP (Mixer Die) exhibited a modulus between these extremes but remained closer to the neat APA curve than to the neat LCP curve rather than aligning with an ideal rule of mixtures midpoint.

The stress at break results (R2~0.971) shows more pronounced nonlinear behavior than the modulus, with deeper curvature and clear crossover behavior at low LCP concentrations. In the LCP content plot, the higher pull speed curve begins near 80–90 MPa, dips slightly at the intermediate compositions, and then rises sharply toward approximately 275 MPa at 100% LCP. The lower pull speed condition shows a more significant decline at intermediate compositions, reaching around 50 MPa near the Mixer Die composition before increasing to approximately 175 MPa for neat LCP. The curvature and crossover observed at lower LCP content suggests competing contribution from the two phases: the ductile APA dominates the strength behavior at low LCP levels, while the increasingly oriented LCP material governs strength as composition approaches 100% LCP. Interestingly, the pull speed alone is not a statistically significant predictor for stress at break (*p* = 0.118), indicating that drawing the neat matrix does not inherently strengthen it. However, the strong interaction term (estimate 124, *p* = 2.20 × 10^−12^) combined with the quadratic LCP effect (estimate 338.0, *p* = 5.54 × 10^−18^) suggests that the peak strength relies heavily on the draw-induced orientation of the reinforcing LCP phase.

Compared to modulus and stress at break, the results for elongation to failure show minimal dependence on pull speed and are primarily governed by material composition. The addition of LCP causes a severe initial drop in elongation, as evidenced by the large negative linear coefficient (estimate −504.2, *p* = 2.35 × 10^−21^), though this loss in ductility is somewhat moderated at higher concentrations by the positive quadratic term. Notably, both the pull speed (*p* = 0.267) and its interaction with the LCP content (*p* = 0.265) are statistically insignificant, meaning the draw ratio has no meaningful impact on the elongation to failure.

#### 3.5.2. Specimens Made with SFE Designs

**Figure 12:**
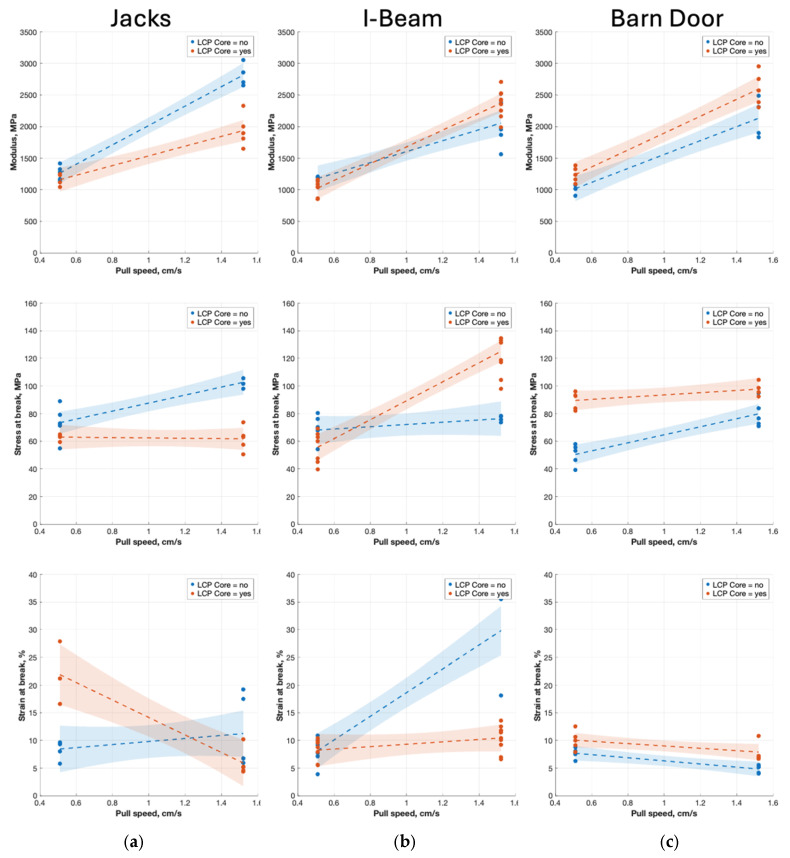
Regression analysis results for the effect of LCP placement and pull speed on the tensile properties of each SFE. (**a**) “Jacks; (**b**) “I-Beam”; (**c**) “Barn Door”.

Regression analysis was also conducted to evaluate how pull speed and material placement influence modulus, stress at break, and elongation to failure for specimens produced with the “Jacks”, “I-Beam”, and “Barn door” SFEs. Model coefficients are provided in Appendix C, Appendix D and Appendix E. with the corresponding responses plotted in Figure 12. The elastic modulus models indicate that stiffness is driven by the pull speed across all three SFE designs, though the effect of material placement varies by geometry. For the “Jacks” design, pull speed has a significant positive effect. The “I-Beam” design sees an increase in modulus with pull speed, but also benefits from a positive interaction when LCP is placed in the core at high speeds. Further, the “Barn Door” design is the only geometry where placing LCP in the core provides a significant independent increase to the modulus, acting additively alongside the strong pull speed effect without a statistically significant interaction between the two.

Stress at break behaviors mirrored these patterns, with material placement interacting with pull speed across the SFE designs. For specimens made with the “Jacks” SFE, the stress at break increased with pull speed but was reduced when the LCP was positioned in the core at high pull speeds. The “I-Beam” SFE produced specimens also exhibiting a crossover behavior. At low pull speeds, LCP placed in the shell was stronger at low pull speeds, but at higher pull speeds, specimens made with LCP in the core were strongest. The fracture surfaces for these strongest specimens showed a stepped morphology and limited fibrillation, indicating progressive LCP material failure with orientation. By comparison, the LCP in the shell configuration showed fewer fibrils at higher speeds. For the “Barn Door” SFE, specimens made with LCP in the core exhibited higher stresses at break across all pull speeds, while placing LCP in the shell resulted in a significant increase with pull speed. Fracture microscopy shows that at low speeds, both configurations failed via brittle LCP sheets with unsheathing from the APA. At higher speeds, specimens made with LCP in the shell displayed increased fibrillation, suggesting partial alignment in high shear regions and a greater strength even though baseline strength is lower than LCP-Core.

Elongation to failure exhibits the most inconsistent and geometry-sensitive behavior among the mechanical properties. For the “I-Beam” SFE, ductility is strongly enhanced by increased pull speed, but this gain is heavily suppressed if the rigid LCP is placed in the core. The “Jacks” SFE design shows the opposite initial trend, where an LCP core independently provides a massive boost to elongation, only for that ductility to be sharply reduced at higher pull speeds due to a strong negative interaction. By comparison, the “Barn Door” SFE design resulted in specimens with the lowest overall baseline ductility and limited sensitivity to material placement and pull speed. Placing the LCP in the core slightly improves elongation, while higher pull speeds slightly reduces it, with no significant interaction between the factors.

In addition to processing and material placement effects, it is important to distinguish between geometric parameters that influence the mechanical performance through different physical mechanisms. The interfacial perimeter provides a measure of the total interface between the immiscible LCP and APA materials and is associated with the increased likelihood of interfacial defects such as delamination or micro flaw initiation, which can reduce the resultant mechanical properties. In contrast, the geometric stress factors reflect the load bearing efficiency of the composite architecture and are governed by the spatial distribution of the reinforcing LCP phase within the cross section. Within the regression framework that was used in this study, these parameters are treated as independent, linear predictors. Their simultaneous statistical significance indicates that they additively contribute to the observed mechanical behavior, rather than through a coupled or nonlinear relationship. This distinction highlights the competing roles of interfacial integrity and structural reinforcement in determining composite performance.

### 3.6. Process–Structure–Property Relations

While evaluating the mechanical performance of individual SFE designs provides valuable insight into specific architectural sensitivities, those independent regression models ultimately treat the die geometry as a categorical variable, limiting their predictive utility for novel designs. To establish a more fundamental understanding, underlying process-structure-property relationships were developed by pooling the experimental data across all SFE geometries. Rather than relying on categorical die labels and initial material placements, this global approach leverages the quantitative structural features extracted directly from the cross-sectional image analysis such as the actual interfacial perimeter, phase-specific moments of inertia, and empirical cross-sectional areas. This methodology isolates the underlying solid mechanics principles governing the composite behavior, demonstrating that structural performance is fundamentally dictated by the physical architecture of the extrudate, regardless of the specific modular die used to generate it. The lateral redistribution of the LCP phase observed across all SFE geometries can be interpreted within the framework for coupled rheological and geometric effects. The lower viscosity and stronger shear thinning behavior of the LCP relative to the APA promotes viscosity driven migration during confined coextrusion. While the SFEs impose spatial constraints on the material arrangement, these constraints do not fully suppress rheology driven redistribution, particularly under high shear and abrupt geometric transitions.

**Figure 13:**
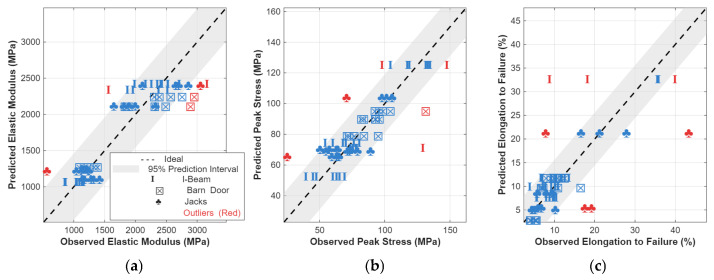
Comparison of (**a**) observed and predicted elastic modulus; (**b**) stress at break; and (**c**) elongation to failure for SFE-made specimens based on observed structures. Distinct markers identify specimens produced using the “I-Beam” (I), “Barn Door” (☒), and “Jacks” (♣) SFE geometries. Blue markers indicate data points (inliers) used to train the final regression models, while red markers represent statistical outliers that were excluded based on a 95% studentized residual threshold. The dashed black line denotes the ideal 1:1 agreement between experimental observations and model predictions.

Appendix F, Appendix G and Appendix H provides the models resulting from best subset selection of all regression models given available predictors as described in Section 3.5. Remarkably, the fidelity of these global models are similar or better than SFE-specific regression models set forth in Appendix C, Appendix D and Appendix E. The correlation plots of Figure 13 show that most of the structural behavior can be explained with relatively few factors such as moments of inertia and interfacial area. Selected global regression models for the elastic modulus, stress at break, and elongation to failure are summarized in Table 5, Table 6 and Table 7, all of which provide R2 values above 0.8.

The global regression model for elastic modulus demonstrates that the composite stiffness is governed by a competition between process-induced orientation and internal geometric complexity. Increased pull speed serves as the primary positive driver for stiffness, adding an estimated 863.8 MPa (*p*-value of 1.66 × 10^−12^) to the modulus at its maximum normalized level. However, this gain is limited by the interfacial perimeter between the LCP and APA phases, which exhibits a strong negative coefficient of −493.6 MPa (*p*-value of 6.47 × 10^−4^). This derived relationship indicates that while drawing the polymers increases orientation, highly complex internal architectures with extensive interfacial areas act as compliance mechanisms that reduce the overall rigidity of the coextruded profile.

Stress at break behavior highlights a severe sensitivity to interfacial flaw generation and sub-optimal material placement. The interfacial perimeter is a highly significant negative predictor (Estimate = −58.94 MPa, *p*-value of 6.26 × 10^−12^), confirming that increasing the surface area between the incompatible polymer melts creates extensive sites for delamination, stress concentration, and premature failure. Furthermore, tensile strength is penalized when the weaker APA matrix dominates the vertical bending axis (APA_Iz_mm4 coefficient of −51.57, *p*-value of 1.11 × 10^−7^) or when the reinforcing LCP material is distributed laterally across the cross-section (Stress_Factor_X coefficient of −44.24, *p*-value of 5.6210^−11^). Also, unlike stiffness, higher pull speeds negatively impact the stress at break in the global model (Estimate = −33.71, *p*-value of 2.51 × 10^−8^), suggesting that higher draw ratios that increase the modulus of the LCP also increase the modulus difference between the LCP and APA, thereby exacerbating dislocations at their interfaces and thus the ultimate strength of the composite.

The elongation to failure model indicates that composite ductility is dictated by physical cross-sectional dimensions and strategic reinforcement placement, as the pull speed factor is statistically insignificant. Preserving a larger total cross-sectional area and a greater mean thickness provides strong positive contributions to ductility, with estimates of 52.22 and 51.29, respectively. Conversely, positioning the highly rigid LCP material to maximize its vertical moment of inertia (LCP_Iz_mm4) results in APA being removed from the outer shell and severe embrittlement of the structure, reducing the elongation limit by a factor of −47.919 at its maximum influence. To maintain a ductile response, the model indicates that the reinforcement should instead be placed laterally within the cross-section, as higher Stress_Factor_X values uniquely contribute to extended failure strains (Estimate = 34.592).

**Figure 14:**
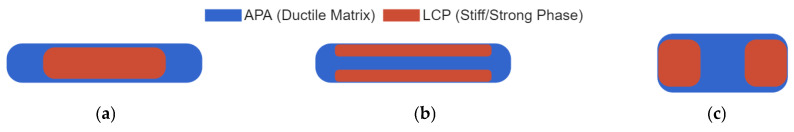
Theoretically optimal coextruded cross-sectional architectures designed to maximize (**a**) elastic modulus; (**b**) stress at break; and (**c**) elongation to failure.

Given these global modeling insights, it is possible to predict optimal architectures as shown in Figure 14 for the elastic modulus (left), stress at break (center), and elongation to failure (right). To ensure a direct comparison of geometric effects, all three profiles set the highly rigid liquid crystalline polymer (LCP, red) material to exactly 50% of the total cross-sectional area within the ductile amorphous polyamide (APA, blue) matrix. The left profile demonstrates the ideal architecture for maximizing elastic modulus featuring a centralized, coaxial LCP core. This configuration minimizes the interfacial perimeter between the two phases, thereby reducing internal compliance and suppressing flaw generation during the high-speed draw-down process. The center profile, designed to maximize stress at break, utilizes a sandwich panel configuration with top and bottom LCP skins. This geometry effectively distributes the strong LCP material to maximize the moment of inertia about the *Z*-axis (IZ) while minimizing the lateral distribution (IX), providing superior resistance to tensile failure. Finally, the right profile highlights the optimal design for maximizing elongation to failure, which relies on a thicker overall cross-section featuring lateral LCP edge-rails. By shifting the stiff LCP material away from the vertical bending axis (minimizing IZ) and concentrating it laterally (maximizing IX), this architecture prevents premature brittle fracture of the core, allowing the ductile APA matrix to govern the extended strain response. Critically, because the geometric requirements for stiffness, strength, and ductility are fundamentally opposed, no single architecture can maximize all three properties, rendering the optimal design highly application dependent.

The improved performance associated with LCP placement in the core can be interpreted in the context of structure processing effects rather than purely geometric considerations. In uniaxial tensile loading along the extrusion direction, the governing mechanisms are related to load path continuity, phase morphology, and interfacial integrity rather than bending dominated structural effects. Core placement modifies the local deformation history experience by the LCP during coextrusion by reducing exposure to wall induced shear gradients. This in turn influences the development of the LCP morphology evolution and phase continuity effects that are captured indirectly through the regression parameters, rather than from geometry alone or bending based structural interpretation.

## 4. Conclusions

This work evaluated a systematic approach for designing shape forming elements (SFEs) to produce architected thermoplastic composites. A dual-method approach utilizing polymer clay prototyping and computational for simulations (ANSYS Polyflow) provided an initial assessment of complex die geometries. The simulations did not successfully predict the macroscopic plastic flow behavior observed during the physical clay prototyping, and they ultimately failed to capture the final material distribution of the liquid crystalline polymer (LCP) and amorphous polyamide (APA) phases in the actual coextrudates. This discrepancy highlights the limitations of relying on simplified flow models to predict the complex viscoelastic interactions, interfacial instabilities, and draw-induced redistributions inherent to multiphase polymer coextrusion.

Cross-sectional and fracture surface microscopy confirmed that the internal composite architecture and subsequent failure mechanisms were highly sensitive to SFE geometry and processing conditions in ways not anticipated by the simulations. Furthermore, material placement and draw ratio directly dictated the structural failure modes. Low pull speeds resulted in brittle fracture and extensive interfacial unsheathing, whereas higher pull speeds induced beneficial fibril formation, stepwise fracture, and enhanced load-bearing capacity within the highly oriented LCP domains.

To move beyond categorical SFE modeling and better understand governing principles, global process-structure-property regression models were developed using all SFE data. These models established that mechanical performance is fundamentally governed by draw-induced orientation coupled with measurable geometric features such as phase-specific moments of inertia, interfacial perimeter, and cross-sectional area. The analysis revealed inherent performance trade-offs: minimizing the interfacial perimeter and centralizing the LCP core maximizes the elastic modulus, whereas maximizing the vertical moment of inertia (Iz) via LCP skins is required to optimize stress at break. Conversely, maximizing elongation to failure demands lateral LCP placement (high IX) to prevent premature brittle fracture of the core. Because the geometric optimizations for stiffness, strength, and ductility are mutually exclusive, there is no single universal architecture; rather, engineers must navigate these structural trade-offs to tailor the final cross-section to specific application requirements.

While the present study was conducted using a single immiscible material system, the governing mechanisms are expected to be broadly applicable to other multiphase thermoplastic systems. In particular, the competition between viscosity contrast, interfacial instability, and geometry enforced confinement is a general feature of coextrusion processing. However, the balance between all these effects, including phase migration and the resulting cross sections, will depend on the specific viscosity ratios, interfacial tensions, and rheological behavior of the selected materials. While the design principles of this work are transferable, the exact performance optimal configurations must be determined for each material system.

The mechanical evaluation in this work was limited to uniaxial tensile loading to directly probe structure-property relationships along the primary processing direction. Other loading modes, such as bending and impact, may activate additional deformation mechanisms including stress gradients, interfacial delamination, and strain rate dependent fracture behavior, and represent important directions for future investigation.

While the fiber-like morphologies observed in the LCP material are directly consistent with flow induced molecular alignment, orientation was not directly quantified in this study using techniques such as X-ray diffraction (XRD) or scanning electron microscopy (SEM). Future work incorporating direct structural characterization methods would further strengthen the correlation between processing conditions, molecular orientation, and mechanical performance in these architected composites.

## Figures and Tables

**Table 1:** Process settings used for all SFEs, including zone temperatures and screw speed.

Process Settings		
Extruder Material	LCP	APA
Die Temperature, °C	250	250
Zone 1, °C	260	250
Zone 2, °C	270	260
Zone 3, °C	240	240
Screw Speed, RPM	5	5
Puller Speeds, cm/s	0.51, 1.52	0.51, 1.52
Chill Roll Temperature, °C	20	20

**Table 2:** Regression model coefficients for elastic modulus as a function of LCP content and pull speed.

Predictor	Estimate	SE	t-Stat	*p*-Value
(Intercept)	976.3	117.0	8.343	1.23 × 10^−9^
PullSpeed	−223.7	108.5	−2.061	0.0472
LCP	−2055	347.8	−5.908	1.27 × 10^−6^
LCP^2	2374	297.1	7.989	3.24 × 10^−9^
LCP*PullSpeed	4015	162.3	24.74	7.04 × 10^−23^

Number of observations: 38, Error degrees of freedom: 33, Root mean squared error: 205, R-squared: 0.991, Adjusted R-squared: 0.99, F-statistic vs. constant model: 910, *p*-value = 2.98 × 10^−33^.

**Table 3:** Regression model coefficients for stress at break as a function of LCP content and pull speed.

Predictor	Estimate	SE	t-Stat	*p*-Value
(Intercept)	97.36	5.096	19.11	9.33 × 10^−20^
PullSpeed	−13.59	8.470	−1.605	0.118
LCP	−267.1	21.72	−12.30	4.55 × 10^−14^
LCP^2	338.0	20.20	16.73	5.54 × 10^−18^
LCP*PullSpeed	124.0	11.63	10.66	2.20 × 10^−12^

Number of observations: 39, Error degrees of freedom: 34, Root mean squared error: 14, R-squared: 0.971, Adjusted R-squared: 0.967, F-statistic vs. constant model: 282, *p*-value = 1.52 × 10^−25^.

**Table 4:** Regression model coefficients for elongation to failure as a function of LCP content and pull speed.

Predictor	Estimate	SE	t-Stat	*p*-Value
(Intercept)	170.0	6.737	24.79	1.86 × 10^−22^
PullSpeed	9.773	8.659	1.129	0.267
LCP	−504.2	22.11	−22.80	2.35 × 10^−21^
LCP^2	349.9	18.87	18.54	1.12 × 10^−18^
LCP*PullSpeed	−12.72	11.21	−1.136	0.265

Number of observations: 37, Error degrees of freedom: 32, Root mean squared error: 12.6, R-squared: 0.963, Adjusted R-squared: 0.959, F-statistic vs. constant model: 210, *p*-value = 1.71 × 10^−22^.

**Table 5:** Regression model coefficients for elastic modulus as a function of structure and process across all SFEs.

Predictor	Estimate	SE	t-Stat	*p*-Value
(Intercept)	1554	116.6	13.33	1.48 × 10^−18^
PullSpeed_Cm_s	863.8	94.33	9.158	1.66 × 10^−12^
Interfacial_Perim_mm	−493.6	136.1	−3.626	6.47 × 10^−4^

Number of observations: 56, Error degrees of freedom: 53, Root mean squared error: 229, R-squared: 0.868, Adjusted R-squared: 0.863, F-statistic vs. constant model: 174, *p*-value = 4.95 × 10^−24^.

**Table 6:** Regression model coefficients for stress at break as a function of structure and process across all SFEs.

Predictor	Estimate	SE	t-Stat	*p*-Value
(Intercept)	162.8	6.945	23.45	2.59 × 10^−29^
PullSpeed_Cm_s	−33.71	5.141	−6.558	2.51 × 10^−8^
Interfacial_Perim_mm	−58.94	6.671	−8.835	6.26 × 10^−12^
APA_Iz_mm4	−51.57	8.385	−6.151	1.11 × 10^−7^
Stress_Factor_X	−44.24	5.378	−8.225	5.62 × 10^−11^

Number of observations: 57, Error degrees of freedom: 52, Root mean squared error: 9.48, R-squared: 0.854, Adjusted R-squared: 0.843, F-statistic vs. constant model: 75.9, *p*-value = 4.55 × 10^−21^.

**Table 7:** Regression model coefficients for elongation to failure as a function of structure and process across SFEs.

Predictor	Estimate	SE	t-Stat	*p*-Value
(Intercept)	−48.049	5.6871	−8.4487	3.392 × 10^−11^
Total_Area_mm2	52.224	7.1236	7.3311	1.8225 × 10^−9^
LCP_Iz_mm4	−47.919	4.3431	−11.033	5.2998 × 10^−15^
Mean_Thickness_mm	51.29	4.5696	11.224	2.8654 × 10^−15^
Stress_Factor_X	34.592	2.3033	15.018	3.6428 × 10^−20^
Inv_Total_Area	46.73	5.775	8.0918	1.1998 × 10^−10^

Number of observations: 56, Error degrees of freedom: 50, Root mean squared error: 2.78, R-squared: 0.835, Adjusted R-squared: 0.818, F-statistic vs. constant model: 50.5, *p*-value = 2.25 × 10^−18^.

**Figure A1:**
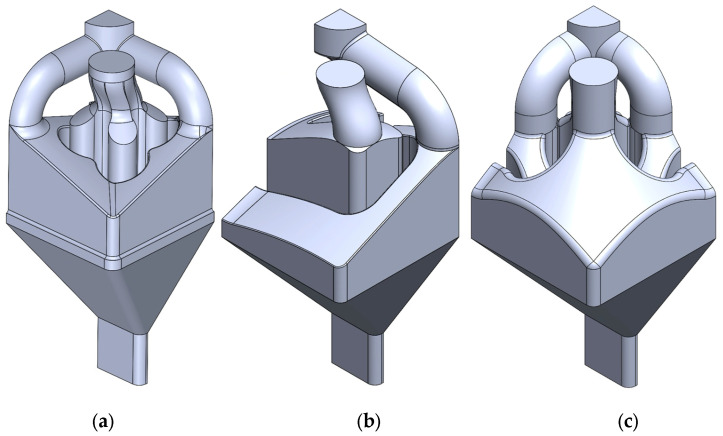
Geometry for SFE simulations (**a**) “Jacks; (**b**) “I-Beam”; and (**c**) “Barn Door”.

## Data Availability

The data are available upon request from the first author.

## Supplementary Materials

The following supporting information can be downloaded at: https://www.mdpi.com/article/10.3390/polym18091098/s1.

## Abbreviations

The following abbreviations are used in this manuscript:

SFE	Shape forming element
LCP	Liquid crystalline polymer
APA	Amorphous polyamide
BCY	Bird–Carreau–Yasuda
FDM	Fused deposition modeling

## Appendix A. Simulation Details

**Table A1:** Fitted BCY parameters used in simulation.

Material	$\eta_{0}$ (Pa.s)	$\eta_{\infty }$ (Pa.s)	λ (s)	*n*
Vectra A950	161.94	0.758 × 10^−6^	0.016	0.370
Trogamid CX7323	588.37	0.222 × 10^−4^	0.002	0.270

## Appendix B. Image Analysis Results

**Table:**

SFE Design & Process	Processed Image & Segmented Image	Evaluated Section Features
SFE_Type: BarnDoor Core_Material: APA Pull_Speed: High		Total_Area_mm2: 6.539 LCP_Area_mm2: 3.359 APA_Area_mm2: 3.180 Interfacial_Perim_mm: 42.10 Total_Iz_mm4: 0.611 LCP_Iz_mm4: 0.321 APA_Iz_mm4: 0.290 y_neutral_mm: 0.753 Total_Ix_mm4: 20.94 LCP_Ix_mm4: 10.60 APA_Ix_mm4: 10.34 x_neutral_mm: 3.286 Total_J_mm4: 21.55 LCP_J_mm4: 10.92 APA_J_mm4: 10.63 Predicted_Modulus_MPa: 3627 Mean_Width_mm: 5.408 Mean_Thickness_mm: 1.017 Aspect_Ratio: 5.316 Stress_Factor_Z: 1.107 Stress_Factor_X: 1.025 Stress_Factor_J: 1.027 Inv_Total_Area: 0.153 Draw_Ratio: 3.059
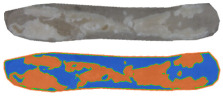
SFE_Type: BarnDoor Core_Material: LCP Pull_Speed: Low		Total_Area_mm2: 13.40 LCP_Area_mm2: 6.717 APA_Area_mm2: 6.687 Interfacial_Perim_mm: 40.64 Total_Iz_mm4: 1.925 LCP_Iz_mm4: 0.904 APA_Iz_mm4: 1.022 y_neutral_mm: 0.912 Total_Ix_mm4: 117.09 LCP_Ix_mm4: 63.02 APA_Ix_mm4: 54.08 x_neutral_mm: 5.612 Total_J_mm4: 119.02 LCP_J_mm4: 63.92 APA_J_mm4: 55.10 Predicted_Modulus_MPa: 3347 Mean_Width_mm: 9.018 Mean_Thickness_mm: 1.287 Aspect_Ratio: 7.009 Stress_Factor_Z: 0.885 Stress_Factor_X: 1.165 Stress_Factor_J: 1.160 Inv_Total_Area: 0.075 Draw_Ratio: 1.492
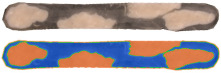
SFE_Type: BarnDoor Core_Material: LCP Pull_Speed: High		Total_Area_mm2: 6.070 LCP_Area_mm2: 3.067 APA_Area_mm2: 3.003 Interfacial_Perim_mm: 33.93 Total_Iz_mm4: 0.639 LCP_Iz_mm4: 0.346 APA_Iz_mm4: 0.292 y_neutral_mm: 0.698 Total_Ix_mm4: 14.55 LCP_Ix_mm4: 7.381 APA_Ix_mm4: 7.171 x_neutral_mm: 2.884 Total_J_mm4: 15.19 LCP_J_mm4: 7.728 APA_J_mm4: 7.463 Predicted_Modulus_MPa: 3712 Mean_Width_mm: 4.874 Mean_Thickness_mm: 1.080 Aspect_Ratio: 4.511 Stress_Factor_Z: 1.185 Stress_Factor_X: 1.029 Stress_Factor_J: 1.035 Inv_Total_Area: 0.165 Draw_Ratio: 3.295
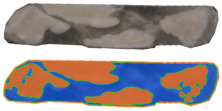
SFE_Type: IBeam Core_Material: APA Pull_Speed: Low		Total_Area_mm2: 9.778 LCP_Area_mm2: 4.916 APA_Area_mm2: 4.862 Interfacial_Perim_mm: 42.26 Total_Iz_mm4: 1.643 LCP_Iz_mm4: 0.792 APA_Iz_mm4: 0.851 y_neutral_mm: 1.061 Total_Ix_mm4: 59.05 LCP_Ix_mm4: 40.95 APA_Ix_mm4: 18.10 x_neutral_mm: 5.276 Total_J_mm4: 60.69 LCP_J_mm4: 41.74 APA_J_mm4: 18.95 Predicted_Modulus_MPa: 3411 Mean_Width_mm: 5.206 Mean_Thickness_mm: 1.139 Aspect_Ratio: 4.568 Stress_Factor_Z: 0.931 Stress_Factor_X: 2.263 Stress_Factor_J: 2.203 Inv_Total_Area: 0.102 Draw_Ratio: 2.045
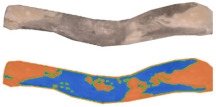
SFE_Type: IBeam Core_Material: APA Pull_Speed: High		Total_Area_mm2: 6.995 LCP_Area_mm2: 3.563 APA_Area_mm2: 3.432 Interfacial_Perim_mm: 27.54 Total_Iz_mm4: 0.792 LCP_Iz_mm4: 0.442 APA_Iz_mm4: 0.351 y_neutral_mm: 0.797 Total_Ix_mm4: 21.26 LCP_Ix_mm4: 16.18 APA_Ix_mm4: 5.078 x_neutral_mm: 3.084 Total_J_mm4: 22.05 LCP_J_mm4: 16.62 APA_J_mm4: 5.429 Predicted_Modulus_MPa: 3787 Mean_Width_mm: 5.199 Mean_Thickness_mm: 1.130 Aspect_Ratio: 4.601 Stress_Factor_Z: 1.259 Stress_Factor_X: 3.186 Stress_Factor_J: 3.061 Inv_Total_Area: 0.143 Draw_Ratio: 2.859
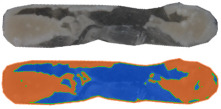
SFE_Type: IBeam Core_Material: LCP Pull_Speed: Low		Total_Area_mm2: 16.39 LCP_Area_mm2: 8.218 APA_Area_mm2: 8.171 Interfacial_Perim_mm: 52.50 Total_Iz_mm4: 4.175 LCP_Iz_mm4: 2.351 APA_Iz_mm4: 1.824 y_neutral_mm: 1.106 Total_Ix_mm4: 115.41 LCP_Ix_mm4: 34.00 APA_Ix_mm4: 81.41 x_neutral_mm: 5.020 Total_J_mm4: 119.59 LCP_J_mm4: 36.35 APA_J_mm4: 83.24 Predicted_Modulus_MPa: 3816 Mean_Width_mm: 8.725 Mean_Thickness_mm: 1.706 Aspect_Ratio: 5.115 Stress_Factor_Z: 1.289 Stress_Factor_X: 0.418 Stress_Factor_J: 0.437 Inv_Total_Area: 0.061 Draw_Ratio: 1.220
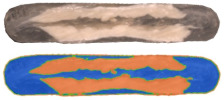
SFE_Type: IBeam Core_Material: LCP Pull_Speed: High		Total_Area_mm2: 5.905 LCP_Area_mm2: 3.004 APA_Area_mm2: 2.901 Interfacial_Perim_mm: 22.90 Total_Iz_mm4: 0.825 LCP_Iz_mm4: 0.621 APA_Iz_mm4: 0.204 y_neutral_mm: 0.885 Total_Ix_mm4: 9.908 LCP_Ix_mm4: 3.999 APA_Ix_mm4: 5.910 x_neutral_mm: 2.514 Total_J_mm4: 10.73 LCP_J_mm4: 4.620 APA_J_mm4: 6.114 Predicted_Modulus_MPa: 4762 Mean_Width_mm: 4.051 Mean_Thickness_mm: 1.246 Aspect_Ratio: 3.250 Stress_Factor_Z: 3.039 Stress_Factor_X: 0.677 Stress_Factor_J: 0.756 Inv_Total_Area: 0.169 Draw_Ratio: 3.387
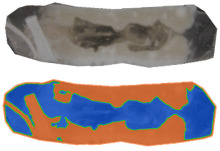
SFE_Type: Jacks Core_Material: APA Pull_Speed: Low		SFE_Type: Jacks Core_Material: APA Pull_Speed: Low Total_Area_mm2: 13.71 LCP_Area_mm2: 6.992 APA_Area_mm2: 6.722 Interfacial_Perim_mm: 49.91 Total_Iz_mm4: 1.994 LCP_Iz_mm4: 0.741 APA_Iz_mm4: 1.252 y_neutral_mm: 1.007 Total_Ix_mm4: 134.30 LCP_Ix_mm4: 60.83 APA_Ix_mm4: 73.48 x_neutral_mm: 5.887 Total_J_mm4: 136.30 LCP_J_mm4: 61.57 APA_J_mm4: 74.73 Predicted_Modulus_MPa: 2860 Mean_Width_mm: 9.665 Mean_Thickness_mm: 1.235 Aspect_Ratio: 7.829 Stress_Factor_Z: 0.592 Stress_Factor_X: 0.828 Stress_Factor_J: 0.824 Inv_Total_Area: 0.073 Draw_Ratio: 1.458
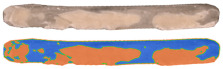
FE_Type: Jacks Core_Material: APA Pull_Speed: High		Total_Area_mm2: 8.516 LCP_Area_mm2: 4.299 APA_Area_mm2: 4.217 Interfacial_Perim_mm: 24.03 Total_Iz_mm4: 1.098 LCP_Iz_mm4: 0.647 APA_Iz_mm4: 0.451 y_neutral_mm: 0.917 Total_Ix_mm4: 33.22 LCP_Ix_mm4: 19.16 APA_Ix_mm4: 14.06 x_neutral_mm: 3.947 Total_J_mm4: 34.32 LCP_J_mm4: 19.80 APA_J_mm4: 14.51 Predicted_Modulus_MPa: 3947 Mean_Width_mm: 6.431 Mean_Thickness_mm: 1.182 Aspect_Ratio: 5.439 Stress_Factor_Z: 1.436 Stress_Factor_X: 1.362 Stress_Factor_J: 1.364 Inv_Total_Area: 0.117 Draw_Ratio: 2.348
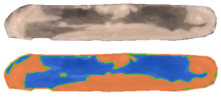
SFE_Type: Jacks Core_Material: LCP Pull_Speed: Low		Total_Area_mm2: 13.53 LCP_Area_mm2: 6.785 APA_Area_mm2: 6.740 Interfacial_Perim_mm: 43.07 Total_Iz_mm4: 2.561 LCP_Iz_mm4: 0.904 APA_Iz_mm4: 1.657 y_neutral_mm: 0.959 Total_Ix_mm4: 93.28 LCP_Ix_mm4: 48.95 APA_Ix_mm4: 44.34 x_neutral_mm: 5.159 Total_J_mm4: 95.85 LCP_J_mm4: 49.85 APA_J_mm4: 45.99 Predicted_Modulus_MPa: 2765 Mean_Width_mm: 7.881 Mean_Thickness_mm: 1.426 Aspect_Ratio: 5.528 Stress_Factor_Z: 0.545 Stress_Factor_X: 1.104 Stress_Factor_J: 1.084 Inv_Total_Area: 0.074 Draw_Ratio: 1.479
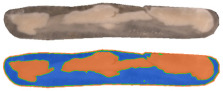
SFE_Type: Jacks Core_Material: LCP Pull_Speed: High		Total_Area_mm2: 9.164 LCP_Area_mm2: 4.606 APA_Area_mm2: 4.557 Interfacial_Perim_mm: 41.03 Total_Iz_mm4: 1.352 LCP_Iz_mm4: 0.494 APA_Iz_mm4: 0.858 y_neutral_mm: 0.878 Total_Ix_mm4: 37.10 LCP_Ix_mm4: 12.97 APA_Ix_mm4: 24.13 x_neutral_mm: 4.403 Total_J_mm4: 38.45 LCP_J_mm4: 13.46 APA_J_mm4: 24.99 Predicted_Modulus_MPa: 2826 Mean_Width_mm: 6.221 Mean_Thickness_mm: 1.270 Aspect_Ratio: 4.899 Stress_Factor_Z: 0.575 Stress_Factor_X: 0.537 Stress_Factor_J: 0.539 Inv_Total_Area: 0.109 Draw_Ratio: 2.183
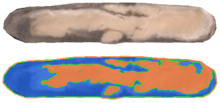

## Appendix C. Regression Models for Specimens Made with “Jacks” SFE Design

**Table A2:** Elastic modulus model (Adj R^2^ = 0.931).

Predictor	Estimate	SE	t-Stat	*p*-Value
(Intercept)	1267	79.00	16.03	2.10 × 10^−10^
LCPCore	−106.3	118.5	−0.8974	0.385
PullSpeed	1550	118.5	13.08	3.07 × 10^−9^
LCPCore*PullSpeed	−773.0	167.6	−4.613	4.03 × 10^−4^

Number of observations: 18, Error degrees of freedom: 14, Root mean squared error: 177, R-squared: 0.943, Adjusted R-squared: 0.931, F-statistic vs. constant model: 77.7, *p*-value = 5.74 × 10^−9^.

**Table A3:** Stress at break model (Adj R^2^ = 0.784).

Predictor	Estimate	SE	t-Stat	*p*-Value
(Intercept)	73.47	3.739	19.65	1.37 × 10^−11^
LCPCore	−10.42	5.608	−1.859	0.0842
PullSpeed	29.25	5.608	5.215	1.31 × 10^−4^
LCPCore*PullSpeed	−30.52	7.931	−3.848	0.00177

Number of observations: 18, Error degrees of freedom: 14, Root mean squared error: 8.36, R-squared: 0.822, Adjusted R-squared: 0.784, F-statistic vs. constant model: 21.6, *p*-value = 1.62 × 10^−5^.

**Table A4:** Elongation to failure model (Adj R^2^ = 0.657).

Predictor	Estimate	SE	t-Stat	*p*-Value
(Intercept)	8.453	1.963	4.306	7.25 × 10^−4^
LCPCore	13.44	3.206	4.194	9.01 × 10^−4^
PullSpeed	2.781	2.776	1.002	0.333
LCPCore*PullSpeed	−18.76	4.241	−4.425	5.77 × 10^−4^

Number of observations: 18, Error degrees of freedom: 14, Root mean squared error: 4.39, R-squared: 0.657, Adjusted R-squared: 0.584, F-statistic vs. constant model: 8.95, *p*-value = 0.00146.

## Appendix D. Regression Models for Specimens Made with “I-Beam” SFE Design

**Table A5:** Elastic modulus model (Adj R^2^ = 0.882).

Predictor	Estimate	SE	t-Stat	*p*-Value
(Intercept)	746.0	156.3	4.709	1.07 × 10^−4^
LCPCore	−388.3	203.7	−1.907	0.0697
PullSpeed	868.4	137.9	6.299	2.43 × 10^−6^
LCPCore*PullSpeed	463.6	176.3	2.630	0.0153

Number of observations: 26, Error degrees of freedom: 22, Root mean squared error: 220, R-squared: 0.896, Adjusted R-squared: 0.882, F-statistic vs. constant model: 63.4, *p*-value = 5.46 × 10^−11^.

**Table A6:** Stress at break model (Adj R^2^ = 0.861).

Predictor	Estimate	SE	t-Stat	*p*-Value
(Intercept)	68.17	4.942	13.79	1.31 × 10^−12^
LCPCore	−12.49	6.735	−1.855	0.0765
PullSpeed	8.242	7.814	1.055	0.302
LCPCore*PullSpeed	61.20	9.830	6.225	2.37 × 10^−6^

Number of observations: 27, Error degrees of freedom: 23, Root mean squared error: 12.1, R-squared: 0.877, Adjusted R-squared: 0.861, F-statistic vs. constant model: 54.5, *p*-value = 1.3 × 10^−10^.

**Table A7:** Elongation to failure model (Adj R^2^ = 0.762).

Predictor	Estimate	SE	t-Stat	*p*-Value
(Intercept)	8.153	1.526	5.343	2.304 × 10^−5^
LCPCore	0.09204	2.079	0.04436	0.965
PullSpeed	21.67	2.643	8.197	3.93 × 10^−8^
LCPCore*PullSpeed	−19.47	3.222	−6.044	4.40 × 10^−6^

Number of observations: 26, Error degrees of freedom: 22, Root mean squared error: 3.74, R-squared: 0.79, Adjusted R-squared: 0.762, F-statistic vs. constant model: 27.7, *p*-value = 1.19 × 10^−7^.

## Appendix E. Regression Models for Specimens Made with “Barn Door” SFE Design

**Table A8:** Elastic modulus model (Adj R^2^ = 0.909).

Predictor	Estimate	SE	t-Stat	*p*-Value
(Intercept)	1018	94.12	10.82	1.76 × 10^−8^
LCPCore	222.6	133.1	1.672	0.0115
PullSpeed	1114	141.2	7.891	1.019 × 10^−6^
LCPCore*PullSpeed	239.4	194.0	1.234	0.236

Number of observations: 19, Error degrees of freedom: 15, Root mean squared error: 210, R-squared: 0.924, Adjusted R-squared: 0.909, F-statistic vs. constant model: 61, *p*-value = 1.23 × 10^−8^.

**Table A9:** Stress at break model (Adj R^2^ = 0.852).

Predictor	Estimate	SE	t-Stat	*p*-Value
(Intercept)	50.47	3.367	15.00	1.94 × 10^−10^
LCPCore	39.13	4.759	8.222	6.14 × 10^−7^
PullSpeed	29.37	4.759	6.172	1.79 × 10^−5^
LCPCore*PullSpeed	−21.20	6.937	−3.056	0.00800

Number of observations: 19, Error degrees of freedom: 15, Root mean squared error: 7.52, R-squared: 0.877, Adjusted R-squared: 0.852, F-statistic vs. constant model: 35.5, *p*-value = 4.66 × 10^−7^.

**Table A10:** Elongation to failure model (Adj R^2^ = 0.644).

Predictor	Estimate	SE	t-Stat	*p*-Value
(Intercept)	7.676	0.6197	12.39	2.80 × 10^−9^
LCPCore	2.366	0.8763	2.700	0.0165
PullSpeed	−2.838	0.8763	−3.239	0.00551
LCPCore*PullSpeed	0.6948	1.278	0.5439	0.595

Number of observations: 19, Error degrees of freedom: 15, Root mean squared error: 1.39, R-squared: 0.704, Adjusted R-squared: 0.644.

## Appendix F. Combinatoric Best Subset Models for Elastic Modulus (MPa)

**Table A11:** One-factor model (Adj R^2^ = 0.846).

Predictor	Estimate	SE	t-Stat	*p*-Value
(Intercept)	1163.9	49.051	23.728	3.0894 × 10^−30^
PullSpeed_Cm_s	1165.2	67.016	17.386	8.7966 × 10^−24^

Number of observations: 56, Error degrees of freedom: 54, Root mean squared error: 250, R-squared: 0.848, Adjusted R-squared: 0.846, F-statistic vs. constant model: 302, *p*-value = 8.8 × 10^−24^.

**Table A12:** Two-factor model (Adj R^2^ = 0.868).

Predictor	Estimate	SE	t-Stat	*p*-Value
(Intercept)	1554	116.6	13.33	1.48 × 10^−18^
PullSpeed_Cm_s	863.8	94.33	9.158	1.66 × 10^−12^
Interfacial_Perim_mm	−493.6	136.1	−3.626	6.47 × 10^−4^

Number of observations: 56, Error degrees of freedom: 53, Root mean squared error: 229, R-squared: 0.868, Adjusted R-squared: 0.863, F-statistic vs. constant model: 174, *p*-value = 4.95 × 10^−24^.

**Table A13:** Three-factor model (Adj R^2^ = 0.880).

Predictor	Estimate	SE	t-Stat	*p*-Value
(Intercept)	1511.9	117.38	12.88	1.1746 × 10^−17^
PullSpeed_Cm_s	1062.5	78.412	13.55	1.5724 × 10^−18^
Mean_Thickness_mm	−531.26	161.87	−3.282	0.0018642
Stress_Factor_X	−256.87	135.59	−1.8945	0.063837

Number of observations: 55, Error degrees of freedom: 51, Root mean squared error: 229, R-squared: 0.886, Adjusted R-squared: 0.88, F-statistic vs. constant model: 133, *p*-value = 4.51 × 10^−24^.

**Table A14:** Four-factor model (Adj R^2^ = 0.884).

Predictor	Estimate	SE	t-Stat	*p*-Value
(Intercept)	1561	119.8	13.03	7.52 × 10^−18^
PullSpeed_Cm_s	1139	79.67	14.17	2.57 × 10^−19^
LCP_Iz_mm4	724.8	312.8	2317	0.0245
Mean_Thickness_mm	−1242	343.5	−3.615	6.88 × 10^−4^
Stress_Factor_X	−329.0	140.3	−2.346	0.0229

Number of observations: 56, Error degrees of freedom: 51, Root mean squared error: 230, R-squared: 0.892, Adjusted R-squared: 0.884, F-statistic vs. constant model: 106, *p*-value = 5.11 × 10^−24^.

**Table A15:** Five-factor model (Adj R^2^ = 0.900).

Predictor	Estimate	SE	t-Stat	*p*-Value
(Intercept)	1364.0	121.5	11.227	2.8423 × 10^−15^
PullSpeed_Cm_s	1218.3	76.655	15.894	3.4683 × 10^−21^
LCP_Iz_mm4	1239.9	297.32	4.1704	0.00012078
Mean_Thickness_mm	−2088.8	357.25	−5.8469	3.7821 × 10^−7^
Stress_Factor_X	−441.92	135.22	−3.2681	0.0019613
Section_Thickness	644.69	141.52	4.5556	3.3801 × 10^−5^

Number of observations: 56, Error degrees of freedom: 50, Root mean squared error: 214, R-squared: 0.909, Adjusted R-squared: 0.9, F-statistic vs. constant model: 100, *p*-value = 7.48 × 10^−25^.

**Table A16:** Six-factor model (Adj R^2^ = 0.926).

Predictor	Estimate	SE	t-Stat	*p*-Value
(Intercept)	1464	130.1	11.26	6.17 × 10^−15^
Total_Area_mm2	−9219	905.4	−10.18	1.78 × 10^−13^
LCP_Iz_mm4	3219	360.8	8.921	1.11 × 10^−11^
APA_Iz_mm4	3259	530.0	6.144	1.62 × 10^−7^
Aspect_Ratio	3407.5	371.4	9.175	4.78 × 10^−12^
Stress_Factor_J	−411.9	103.1	−3.996	2.25 × 10^−4^
Section_Thickness	1152.4	143.6	8.028	2.315 × 10^−11^

Number of observations: 54, Error degrees of freedom: 47, Root mean squared error: 186, R-squared: 0.934, Adjusted R-squared: 0.926, F-statistic vs. constant model: 111, *p*-value = 4.64 × 10^−26^.

## Appendix G. Combinatoric Best Subset Models for Stress at Break (MPa)

**Table A17:** One-factor model (Adj R^2^ = 0.675).

Predictor	Estimate	SE	t-Stat	*p*-Value
(Intercept)	67.172	2.4638	27.264	1.2434 × 10^−33^
Stress_Factor_Z	58.012	5.3528	10.838	2.8913 × 10^−15^

Number of observations: 57, Error degrees of freedom: 55, Root mean squared error: 13.5, R-squared: 0.681, Adjusted R-squared: 0.675, F-statistic vs. constant model: 117, *p*-value = 2.89 × 10^−15^.

**Table A18:** Two-factor model (Adj R^2^ = 0.750).

Predictor	Estimate	SE	t-Stat	*p*-Value
(Intercept)	91.01	5.634	16.15	2.49 × 10^−22^
Interfacial_Perim_mm	−36.80	6.961	−5.287	2.30 × 10^−6^
Stress_Factor_Z	35.38	7.40	4.781	1.39 × 10^−5^

Number of observations: 57, Error degrees of freedom: 54, Root mean squared error: 12.7, R-squared: 0.759, Adjusted R-squared: 0.750, F-statistic vs. constant model: 85.2, *p*-value = 1.97 × 10^−17^.

**Table A19:** Three-factor model (Adj R^2^ = 0.832).

Predictor	Estimate	SE	t-Stat	*p*-Value
(Intercept)	93.67	6.338	14.83	1.68 × 10^−20^
Interfacial_Perim_mm	−32.86	7.287	−4.509	3.63 × 10^−5^
Stress_Factor_X	−1342	237.8	−5.644	6.63 × 10^−7^
Stress_Factor_Z	1330	240.8	5.523	1.03 × 10^−6^

Number of observations: 57, Error degrees of freedom: 53, Root mean squared error: 10.4, R-squared: 0.841, Adjusted R-squared: 0.832, F-statistic vs. constant model: 93.7, *p*-value = 3.47 × 10^−21^.

**Table A20:** Four-factor model (Adj R^2^ = 0.843).

Predictor	Estimate	SE	t-Stat	*p*-Value
(Intercept)	162.8	6.945	23.45	2.59 × 10^−29^
PullSpeed_Cm_s	−33.71	5.141	−6.558	2.51 × 10^−8^
Interfacial_Perim_mm	−58.94	6.671	−8.835	6.26 × 10^−12^
APA_Iz_mm4	−51.57	8.385	−6.151	1.11 × 10^−7^
Stress_Factor_X	−44.24	5.378	−8.225	5.62 × 10^−11^

Number of observations: 57, Error degrees of freedom: 52, Root mean squared error: 9.48, R-squared: 0.854, Adjusted R-squared: 0.843, F-statistic vs. constant model: 75.9, *p*-value = 4.55 × 10^−21^.

**Table A21:** Five-factor model (Adj R^2^ = 0.874).

Predictor	Estimate	SE	t-Stat	*p*-Value
(Intercept)	162.8	6.798	23.95	4.71 × 10^−29^
PullSpeed_Cm_s	−38.02	5.143	−7.392	1.47 × 10^−9^
Interfacial_Perim_mm	−52.61	8.095	−6.499	3.65 × 10^−8^
APA_Iz_mm4	−76.75	15.09	−5.085	5.53 × 10^−6^
Mean_Thickness_mm	21.45	11.59	1.851	0.070
Stress_Factor_X	−38.99	5.790	−6.734	1.56 × 10^−8^

Number of observations: 56, Error degrees of freedom: 50, Root mean squared error: 9.01, R-squared: 0.885, Adjusted R-squared: 0.874, F-statistic vs. constant model: 77, *p*-value = 2.72 × 10^−22^.

**Table A22:** Six-factor model (Adj R^2^ = 0.882).

Predictor	Estimate	SE	t-Stat	*p*-Value
(Intercept)	146.11	18.685	7.8194	3.1671 × 10^−11^
Total_Area_mm2	145.56	39.955	3.6432	0.00063956
Interfacial_Perim_mm	−122.16	32.749	−3.7302	0.00048912
Mean_Thickness_mm	−79.27	10.284	−7.7083	4.7106 × 10^−10^
Stress_Factor_Z	48.262	7.49	6.4435	4.4545 × 10^−8^
Stress_Factor_X	−32.67	5.4371	−6.0086	2.1224 × 10^−7^
Section_Thickness	−63.601	26.919	−2.3627	0.022076

Number of observations: 57, Error degrees of freedom: 50, Root mean squared error: 8.67, R-squared: 0.895, Adjusted R-squared: 0.882, F-statistic vs. constant model: 70.9, *p*-value = 9.91 × 10^−23^.

## Appendix H. Combinatoric Best Subset Models for Strain at Break (%)

**Table A23:** One-factor model (Adj R^2^ = 0.105).

Predictor	Estimate	SE	t-Stat	*p*-Value
(Intercept)	6.7265	1.0195	6.5981	1.5933 × 10^−8^
Section_Thickness	4.6073	1.6598	2.7758	0.0074733

Number of observations: 58, Error degrees of freedom: 56, Root mean squared error: 3.72, R-squared: 0.121, Adjusted R-squared: 0.105, F-statistic vs. constant model: 7.7, *p*-value = 0.00747.

**Table A24:** Two-factor model (Adj R^2^ = 0.247).

Predictor	Estimate	SE	t-Stat	*p*-Value
(Intercept)	11.356	0.92791	12.238	1.8473 × 10^−17^
Interfacial_Perim_mm	−12.007	2.673	−4.4919	3.5784 × 10^−5^
APA_Iz_mm4	11.123	2.6135	4.2558	8.009 × 10^−5^

Number of observations: 59, Error degrees of freedom: 56, Root mean squared error: 3.98, R-squared: 0.273, Adjusted R-squared: 0.247, F-statistic vs. constant model: 10.5, *p*-value = 0.000131.

**Table A25:** Three-factor model (Adj R^2^ = 0.438).

Predictor	Estimate	SE	t-Stat	*p*-Value
(Intercept)	17.458	1.8105	9.6425	3.5989 × 10^−13^
Interfacial_Perim_mm	−21.182	3.2213	−6.5758	2.3486 × 10^−8^
APA_Iz_mm4	14.189	2.3875	5.9431	2.3681 × 10^−7^
Stress_Factor_Z	−6.6836	2.1527	−3.1047	0.0030797

Number of observations: 56, Error degrees of freedom: 52, Root mean squared error: 3.49, R-squared: 0.469, Adjusted R-squared: 0.438, F-statistic vs. constant model: 15.3, *p*-value = 2.96 × 10^−7^.

**Table A26:** Four-factor model (Adj R^2^ = 0.566).

Predictor	Estimate	SE	t-Stat	*p*-Value
(Intercept)	−9.736	2.938	−3.314	0.00165
Total_Area_mm2	−43.01	7.502	−5.733	4.54 × 10^−7^
Mean_Thickness_mm	50.59	8.059	6.274	6.19 × 10^−8^
Aspect_Ratio	28.92	5.779	5.005	6.29 × 10^−6^
Stress_Factor_X	26.11	2.960	8.821	4.78 × 10^−12^

Number of observations: 59, Error degrees of freedom: 54, Root mean squared error: 4.08, R-squared: 0.596, Adjusted R-squared: 0.566, F-statistic vs. constant model: 19.9, *p*-value = 3.97 × 10^−10^.

**Table A27:** Five-factor model (Adj R^2^ = 0.818).

Predictor	Estimate	SE	t-Stat	*p*-Value
(Intercept)	−48.049	5.6871	−8.4487	3.392 × 10^−11^
Total_Area_mm2	52.224	7.1236	7.3311	1.8225 × 10^−9^
LCP_Iz_mm4	−47.919	4.3431	−11.033	5.2998 × 10^−15^
Mean_Thickness_mm	51.29	4.5696	11.224	2.8654 × 10^−15^
Stress_Factor_X	34.592	2.3033	15.018	3.6428 × 10^−20^
Inv_Total_Area	46.73	5.775	8.0918	1.1998 × 10^−10^

Number of observations: 56, Error degrees of freedom: 50, Root mean squared error: 2.78, R-squared: 0.835, Adjusted R-squared: 0.818, F-statistic vs. constant model: 50.5, *p*-value = 2.25 × 10^−18^.

**Table A28:** Six-factor model (Adj R^2^ = 0.849).

Predictor	Estimate	SE	t-Stat	*p*-Value
(Intercept)	−51.01	6.186	−9.862	3.19 × 10^−13^
PullSpeed_Cm_s	6.176	1.843	3.352	0.00155
Total_Area_mm2	72.03	8.179	8.806	1.15 × 10^−11^
LCP_Iz_mm4	−48.75	4.049	−12.04	2.98 × 10^−16^
Mean_Thickness_mm	45.81	4.270	10.73	1.82 × 10^−14^
Stress_Factor_X	37.54	2.273	16.52	1.16 × 10^−21^
Inv_Total_Area	54.02	5.643	9.572	8.40 × 10^−13^

Number of observations: 56, Error degrees of freedom: 49, Root mean squared error: 2.53, R-squared: 0.865, Adjusted R-squared: 0.849, F-statistic vs. constant model: 52.5, *p*-value = 1.16 × 10^−19^.
